# Construction of Binary Locally Repairable Codes with Nonuniform Locality and Availability Using Combinatorial Designs

**DOI:** 10.3390/e27030269

**Published:** 2025-03-05

**Authors:** Yu Zhang, Xiangqiong Zeng

**Affiliations:** 1 School of Information Management, Shanghai Lixin University of Accounting and Finance, Shanghai 201209, China; 2 School of Materials and Chemistry, University of Shanghai for Science and Technology, Shanghai 200093, China; zengxq202312@163.com

**Keywords:** coding theory, distributed storage, pairwise balanced designs

## Abstract

In this paper, we generalize the construction of locally repairable codes (LRCs) by leveraging pairwise balanced designs (PBDs) and balanced incomplete block designs (BIBDs) to construct codes with nonuniform locality or nonuniform availability. Our constructions prioritize binary implementations for practical deployment while achieving optimal or near-optimal performance in terms of rate, minimum distance, and repair efficiency. Specifically, we propose distance-optimal LRCs with nonuniform localities and message-symbol (r,t)-availability. These binary constructions achieve optimal minimum distance under known bounds and have higher code rates than existing works. We also address open problems in the literature, including constructions where r∤k, and demonstrate that our constructions encompass or outperform several prior works.

## 1. Introduction

Large-scale distributed storage systems store vast amounts of data, which are vulnerable to loss in the event of node failures. A widely adopted method to prevent data loss is the replication mechanism, where data from a failed node are recovered by copying it from one of its replicas to a new replacement node. This approach ensures data integrity and system reliability but introduces significant storage overhead. To address this inefficiency, erasure codes have been proposed as an alternative to replication, offering reduced storage overhead and the same level of reliability. Facebook’s cluster storage system [1] employs a (14,10) Reed–Solomon (RS) code. This system can tolerate up to four node failures. However, such codes require all the data from the other 10 nodes to repair a single node. Therefore, there is a critical need for coding schemes that minimize the number of nodes accessed during recovery. To address this problem, locally repairable codes (LRCs) [2] have been introduced.

In recent years, locally repairable codes have gained significant attention due to their practical applications in distributed storage systems [2,3,4,5]. The primary objective of LRCs is to minimize the number of nodes that need to be accessed when repairing a failed node. In [4], the concept of [n,k,d] LRCs with (r,δ)-locality was introduced. Cai et al. [6] gave a construction of locally repairable codes with (r,δ)-locality and smaller field sizes than known constructions of this kind.

However, these constructions do not support parallel reading of specific data nodes. In practice, some data experience significantly higher access frequencies, and such data are called hot data. For such data, supporting parallel access is an important feature. To address this shortcoming, Wang et al. [7] introduced ${\left(n,k,r,t\right)}_{q}$. These codes enable the retrieval of data stored in an individual node using t+1 mutually exclusive recovery sets, thereby enabling parallel access to frequently accessed data in distributed storage systems. Below, we give a formal definition of ${\left(n,k,r,t\right)}_{q}$ LRC.

**Definition 1.**  *Given a systematic ${\left[n,k,d\right]}_{q}$ linear code C with block length n, dimension k, and minimum distance d over $F_{q}$, where d(C) denotes the minimum distance of C, R(C) denotes the code rate of C, the k×n generator matrix of C is defined as $G=[I_{k}|P]$, and the (n−k)×n parity-check matrix of C is defined as $H=[-P|I_{n-k}]$. A message m of length k over $F_{q}$ is encoded into a codeword c of length n over $F_{q}$. The encoding process can be written in the form of matrix multiplication: mG=c.*

*Let [n] denote the set {1,…,n}. A symbol $c_{i}$ in a codeword c∈C has locality r if $c_{i}$ is a linear combination of a subset of c indexed by R(i)∈[n]∖{i}, where |R(i)|≤r. R(i) is a recovery set (repair set) of $c_{i}$. C is said to have all-symbol locality if every symbol in C has locality r.*

*If a symbol $c_{i}$ in a codeword c∈C has t repair sets $R_{1}\left(i\right),\ldots ,R_{t}\left(i\right)\in \left[n\right]\setminus \left\{i\right\}$, where $|R_{i}\left(i\right)|\leq r,\;j\in \left[t\right]$, $R_{l}\left(i\right)\cap R_{m}\left(i\right)\;\forall l\neq m$, then the symbol $c_{i}$ has locality r and availability t, or the symbol has (r,t)-availability [8].*

*For an [n,k,d] code C over $F_{q}$, if all information symbols possess (r,t)-availability, C is called an ${\left(n,k,r,t\right)}_{q}$ locally repairable code (LRC) with information-symbol (IS) (r,t)-availability. This type of code is abbreviated as IS-LRC. Alternatively, if every codeword symbol in C has (r,t)-availability, C is referred to as an ${\left(n,k,r,t\right)}_{q}$ LRC with all-symbol (r,t)-availability, abbreviated as AS-LRC.*

The repair sets of any coordinate *i* in a codeword c from an (n,k,r,t) locally repairable code can be deduced from its parity-check matrix *H*. Let $h_{1},\ldots ,h_{n-k}$ denote the rows of *H*. If coordinate *i* possesses (r,t)-availability, then the *i*-th column of *H* has at least *t* nonzero elements in *t* rows $h_{i_{1}},\ldots ,h_{i_{t}}$. Note that $cH^{T}=0$. So, we can obtain *t* linear equations $c\cdot h_{i_{1}}=0,\ldots ,c\cdot h_{i_{t}}=0$. Assume $c_{i}$ is lost. Then, the terms in the above *t* linear equations, other than $c_{i}$, index a repair set of $c_{i}$.

**Example 1.**  *G is a generator matrix of an ${\left(8,4,2,2\right)}_{7}$ LRC C over $F_{7}$. H is its parity-check matrix.*

(1) $$G=\left(\begin{array}{cccccccccc} 1 & 0 & 0 & 0 & 1 & 0 & 1 & 0 & 6 & 2\\ 0 & 1 & 0 & 0 & 1 & 0 & 0 & 1 & 2 & 2\\ 0 & 0 & 1 & 0 & 0 & 1 & 1 & 0 & 2 & 5\\ 0 & 0 & 0 & 1 & 0 & 1 & 0 & 1 & 5 & 6 \end{array}\right),H=\left(\begin{array}{cccccccccc} 6 & 6 & 0 & 0 & 1 & 0 & 0 & 0 & 0 & 0\\ 0 & 0 & 6 & 6 & 0 & 1 & 0 & 0 & 0 & 0\\ 6 & 0 & 6 & 0 & 0 & 0 & 1 & 0 & 0 & 0\\ 0 & 6 & 0 & 6 & 0 & 0 & 0 & 1 & 0 & 0\\ 1 & 5 & 5 & 2 & 0 & 0 & 0 & 0 & 1 & 0\\ 5 & 5 & 2 & 1 & 0 & 0 & 0 & 0 & 0 & 1 \end{array}\right).$$

*In this code C, the message symbols $c_{1},c_{2},c_{3},c_{4}$ in any codeword c can be recovered by two disjoint repair sets of size 2. Assume $c_{1}$ is lost. From H or G, we can see that $c_{1}+c_{2}=c_{5}$ and $c_{1}+c_{3}=c_{7}$. So, the two disjoint repair sets of $c_{1}$ are $R_{1}\left(1\right)=\left\{2,5\right\}$ and $R_{2}\left(1\right)=\left\{3,7\right\}$. So, $c_{1}$ can be recovered by reading the data stored in $c_{2},c_{5}$ (or $c_{3},c_{7}$), and $c_{5}-c_{2}=c_{1}$.*

Li et al. [9] constructed asymptotically good LRCs with multiple recovery sets using automorphism groups of function fields. Wang et al. [7] proposed the following minimum distance bound for ${\left(n,k,r,t\right)}_{q}$ LRC:

(2) $$d\leq n-k-\left\lceil \frac{t(k-1)+1}{t(r-1)+1}\right\rceil +2.$$

Rawat et al. [8] demonstrated that for (n,k,r,t) LRCs, where each repair group includes precisely one parity symbol, the minimum distance *d* adheres to the following relationship:

(3) $$d\leq n-k-\left\lceil \frac{kt}{r}\right\rceil +t+1.$$

In their work, Rawat et al. [8] employed combinatorial designs to develop locally repairable codes with (r,t)-availability under the condition that *r* divides *k*. These constructions were shown to meet the bound in (3). The authors also raised an open question: whether r|k is a necessary condition for (n,k,r,t) locally repairable codes to attain the bound in (3). Su et al. [10] introduced binary LRCs constructed using resolvable configurations. Su [11] gave additional parameters for locally repairable codes whose minimum distance attains the bound (3) based on resolvable configurations.

It is important to highlight that binary codes over $F_{2}$ offer significant benefits for practical applications compared to codes defined over larger fields. Hao et al. [12] presented methods for constructing locally repairable codes from LDPC codes. Balaji et al. [13] gave a definition of the strict availability of locally repairable codes. Zhang et al. [14] extended this construction by relaxing the constraint $n={\left(r+1\right)}^{x},x\geq 2$ given in [13]. Zhang et al. [15] also gave a series of distance-optimal locally repairable codes from certain combinatorial structures to attain the bound in (3). Among the codes proposed in [15], r|k is not a necessary condition. Teng et al. [16] developed constructions of optimal binary locally repairable codes (LRCs) with multiple recovery sets. Jin et al. [17] introduced a new class of binary LRCs with high availability. Tan et al. [18] proposed several constructions of binary distance-optimal LRCs from linear algebra and partial geometries. Fetrat et al. [19] presented a novel family of binary LRCs designed for coded distributed computing.

Prakash et al. [20] derived the following upper bound on the code rate for t=2:

(4) $$\frac{k}{n}\leq \frac{r}{r+2}.$$

In practice, distributed storage systems often involve nodes with different characteristics, such as varying failure rates or uptimes. Existing constructions are not fully equipped to address these heterogeneous requirements. Kadhe et al. [21] and Zeh et al. [22] proposed LRCs with nonuniform localities, while Bhadane et al. [23] introduced LRCs with varying localities and availabilities. Cai et al. [24] derived a connection between (k,R,1)-packing and optimal locally repairable codes. They also proposed a classification of LRCs with nonuniform locality or availability and specific conditions that the parameters need to satisfy under these classifications [24]. Cai et al. [25] generalized the concept of (r,t)-availability and provided a series of constructions that achieve the bound in (3).

This paper generalizes these approaches by leveraging pairwise balanced designs (PBDs) and balanced incomplete block designs (BIBDs) to construct LRCs with nonuniform localities and availabilities. Table 1 presents the parameters of all constructions proposed in this article. These codes prioritize binary implementations for ease of deployment while achieving optimal or near-optimal performance in terms of rate, minimum distance, and repair efficiency. The contributions of this paper are as follows:

1. We introduce LRCs with nonuniform availabilities and all-symbol availabilities from pairwise balanced designs. This construction contains the codes with all-symbol availabilities constructed in [15]. Moreover, from this construction, we obtain a distance-optimal binary (n=7,k=3,r=2,t=2) LRC with all-symbol availability attaining the bounds in (2) and (3);
2. We also give a construction of LRCs with nonuniform localities and all-symbol availability from PBDs. This construction also contains the codes from [15];
3. We introduce LRCs with nonuniform availabilities and uniform locality using PBDs. When the chosen PBD is a BIBD, this construction gives distance-optimal locally repairable codes with message (r,t)-availability. The rate of this construction is optimal when t=2. This construction also contains some codes from [15,18,24], and the rate of this construction is higher than that of the codes in [11,24,25];
4. Finally, we propose distance-optimal LRCs with nonuniform localities using PBDs. This construction gives distance-optimal locally repairable codes with nonuniform localities and message (r,t)-availability attaining the bound in (3). The rate of this construction is higher than the codes in [11,24,25] while requiring a much smaller field size. This construction also contains some codes from [15,18,24]. This construction gives codes with parameters not contained in [11,15,18,24,25].

## 2. Preliminaries

This section outlines the notations and foundational concepts applicable throughout this article. We define the set [i] for any positive integer *i* as 1,2,…,i, and the set [i,j] for i<j as i,i+1,…,j. The symbol $F_{q}$ represents the finite field with *q* elements, where *q* is a prime power. Additionally, the support of a vector x is indicated by suppx.

### 2.1. Block Designs

Let $\hat{v},\hat{k},\hat{\lambda }$ be integers such that $\hat{v}\geq \hat{k}\geq 2$ and $\hat{\lambda }\geq 1$. A finite set *X* is denoted as the point set. Elements in B are subsets of *X*. Elements in B are called blocks. The pair (X,B) is called a *balanced incomplete block design* (BIBD), written as a $\left(\hat{v},\hat{k},\hat{\lambda }\right)$-BIBD, if the following conditions are satisfied: $\left|X\right|=\hat{v}$, $\left|B\right|=\hat{k}$ for every B∈B, and every pair of distinct points in *X* is contained in exactly $\hat{\lambda }$ blocks [26]. In a $\left(\hat{v},\hat{k},\hat{\lambda }\right)$-BIBD, each point belongs to $\hat{r}=\frac{\hat{\lambda }\left(\hat{v}-1\right)}{\hat{k}-1}$ blocks, and the number of blocks is given by $\hat{b}=\frac{\hat{v}\hat{r}}{\hat{k}}=\frac{\hat{\lambda }\left({\hat{v}}^{2}-\hat{v}\right)}{{\hat{k}}^{2}-\hat{k}}$.

A balanced incomplete block design can be described using its incidence matrix. For a $\left(\hat{v},\hat{b},\hat{r},\hat{k},\hat{\lambda }\right)$-BIBD (X,B), where *X* is a point set of size $\hat{v}$ and B is a block set of size $\hat{b}$, let *M* represent a $\hat{v}\times \hat{b}$ incidence matrix. The columns of *M* correspond to the blocks $B_{1},B_{2},\ldots ,B_{\hat{b}}$, with each $B_{i}\in B$ for $i\in [\hat{b}]$. The rows of *M* correspond to the points $p_{1},p_{2},\ldots ,p_{\hat{v}}$. The entry $m_{ij}$ in the incidence matrix *M* is defined as

(5) $$m_{ij}=\left\{\begin{array}{cc} 1, & \mathrm{if}\;p_{j}\in B_{i},\\ 0, & \mathrm{otherwise}. \end{array}\right.$$

In this matrix, each column contains exactly $\hat{k}$ ones, while each row contains exactly $\hat{r}$ ones.

A *resolution* is defined as a partition of B into multiple parallel classes. If such a partition exists, the BIBD is called *resolvable* [26]. The union of all parallel classes is equal to the point set *X*. A $\left(\hat{v},\hat{k},\hat{\lambda }\right)$-RBIBD denotes a resolvable BIBD. The existence of a parallel class implies that $\hat{k}$ divides $\hat{v}$, and the parallel class contains exactly $\hat{v}/\hat{k}$ blocks. For a resolvable BIBD, B can be partitioned into $\hat{b}/\left(\hat{v}/\hat{k}\right)=\hat{r}$ parallel classes.

### 2.2. Pairwise Balanced Designs (PBDs)

A PBD is a generalization of the balanced incomplete block design (BIBD) and is defined below.

Consider a positive integer $\hat{v}$, a set of positive integers $\hat{K}$ such that $\hat{v}\geq \hat{k}$ for all $\hat{k}\in \hat{K}$, and another positive integer $\hat{\lambda }$. Let *X* be a finite set, referred to as the set of points, and let B be a collection of subsets of *X*, known as blocks. The pair (X,B) is defined as a $\left(\hat{v},\hat{K},\hat{\lambda }\right)$-*pairwise balanced design* (PBD) [26], abbreviated as $\left(\hat{v},\hat{K},\hat{\lambda }\right)$-PBD, if it satisfies the following conditions: The cardinality of *X* is $\hat{v}$, i.e., $\left|X\right|=\hat{v}$. For every block B∈B, the size |B| belongs to $\hat{K}$, and any pair of distinct points in *X* appears in exactly $\hat{\lambda }$ blocks.

The set of parameters $\left\{\hat{v},\hat{K},\hat{\lambda }\right\}$ is known as the parameter set of the PBD (X,B). When $\hat{K}$ contains only one element, i.e., $\hat{K}=\left\{\hat{k}\right\}$, the $\left(\hat{v},\hat{K},\hat{\lambda }\right)$-PBD is a $\left(\hat{v},\hat{k},\hat{\lambda }\right)$-BIBD. A $\left(\hat{v},\hat{K},\hat{\lambda }\right)$-PBD (X,B) is considered *nondegenerate* if there exists at least one block B∈B such that $2\leq |B|<\hat{v}$. In this paper, all PBDs discussed are assumed to be nondegenerate. PBDs can also be represented via incidence matrices.

A PBD can be constructed by removing points from a BIBD [26]. Suppose (X,B) is a $\left(\hat{v},\hat{k},\hat{\lambda }\right)$-BIBD, and let p∈X be any point. Define $X_{p}=X\setminus \left\{p\right\}$ as the point set excluding *p*, and let $B_{p}=\left\{B\setminus \left\{p\right\}:B\in B\right\}$ be the collection of blocks obtained by removing *p* from each block in B. Then, the pair $\left(X_{p},B_{p}\right)$ forms a $\left(\hat{v},\left\{\hat{k}-1,\hat{k}\right\},\hat{\lambda }\right)$-PBD.

## 3. Code Construction

### 3.1. Locally Repairable Codes from PBDs with Nonuniform Availability and All-Symbol Locality

First, we construct an LRC with different availability and all-symbol locality using certain special PBDs. Since general PBDs have different block sizes and the occurrence frequency of each point also differs, it is relatively difficult to use general PBDs to construct locally repairable codes. However, certain special PBDs can be selected for constructing locally repairable codes. In this section, we choose a $\left(\hat{v},\hat{K},\hat{\lambda }\right)$-PBD such that the number of appearances of each point is $\hat{r}$.

Consider a $\left(\hat{v},\hat{K},1\right)$-PBD (X,B), where each point in *X* occurs exactly $\hat{r}$ times across the blocks in B, and the total number of blocks is $\left|B\right|=\hat{b}$. In any nondegenerate PBD, it holds that $\hat{b}\geq \hat{v}$. The incidence matrix *M* associated with this PBD can be utilized to construct a locally repairable code. Let us denote this matrix by *H*. Define ${\hat{k}}_{\min}$ as the smallest value in $\hat{K}$, ${\hat{k}}_{\max}$ as the largest value in $\hat{K}$, and ${\hat{k}}_{\mathrm{avg}}$ as the average of all elements in $\hat{K}$.

**Theorem 1.**  *H defines an ${\left(n=\hat{b},k,r=\hat{r}-1,t={\hat{k}}_{\min}\right)}_{2}$ locally repairable code. This code provides $\left(r=\hat{r}-1,t={\hat{k}}_{\min}\right)$-availability, where the code length is $\hat{b}$, the minimum distance satisfies d(C)≥t+1, and the code rate is bounded below by $R\left(C\right)\geq 1-\frac{{\hat{k}}_{\mathrm{avg}}}{r}$.*

**Proof.**  From the earlier construction, it is established that *M* serves as the incidence matrix of a $\left(\hat{v},\hat{K},\hat{\lambda }\right)$-PBD (X,B). For the matrix H=M, its rows are indexed by the points of the PBD, and its columns are indexed by the blocks. Each row of *H* has a weight equal to $\hat{r}$. Unlike LRCs derived from BIBDs, the columns of *H* do not necessarily have uniform weights. Specifically, the smallest column weight of *H* is denoted by ${\hat{k}}_{\min}$, the largest column weight is denoted by ${\hat{k}}_{\max}$, and the average column weight is denoted by ${\hat{k}}_{\mathrm{avg}}$.

In a PBD, any two distinct points appear together in exactly one block. As a result, the supports of the rows of *H* that correspond to different points intersect only at that coordinate. If the supports of these rows were to intersect more than once, it would imply that two distinct points in *X* are present together in more than one block, which violates the fundamental properties of a $\left(\hat{v},\hat{K},\hat{\lambda }\right)$-PBD. The supports of the rows in *H* associated with the same coordinate are allowed to overlap at one coordinate.

When *H* is used as the parity-check matrix of a locally repairable code, each code symbol is associated with at least ${\hat{k}}_{\min}$ different sets. The size of each recovery set is $\hat{r}-1$. It should be emphasized that the number of recovery sets varies across code symbols and is not uniform throughout the code. The minimum number of recovery sets for any symbol is ${\hat{k}}_{\min}$, while the maximum number is ${\hat{k}}_{\max}$.

Since every code symbol has no fewer than ${\hat{k}}_{\min}$ disjoint recovery sets, the parameter *t* can be assigned the value ${\hat{k}}_{\min}$. Consequently, *H* acts as the parity-check matrix.

The parameters of our construction in the theorem are ${\left(n=\hat{b},k,r=\hat{r}-1,t={\hat{k}}_{\min}\right)}_{q}$. We obtain an AS-LRC with nonuniform availability.

We now analyze the parameters of our code. Let c denote a codeword in our construction of a locally repairable code. The weight of c corresponds to the minimum distance *d*. Note that every code symbol has *t* repair sets of size r+1. In this case, any erased symbol in c can be deduced when no more than *t* positions in c are erased. As a result, the minimum distance d(C) satisfies the condition d(C)≥t+1.

Next, we determine the code rate of the proposed construction. The matrix *H* is of size $\hat{v}\times \hat{b}$. Let $m=\hat{v}$ and $n=\hat{b}$. The relationship $m\cdot \hat{r}=n\cdot {\hat{k}}_{\mathrm{avg}}$ holds: rank(H)≤m. Then, the code rate can be computed as follows:

$$\begin{array}{cc} R(C) & =1-\frac{\mathrm{rank}(H)}{n}\\ \; & \geq 1-\frac{m}{n}\\ \; & =1-\frac{{\hat{k}}_{\mathrm{avg}}}{\hat{r}}\\ \; & =1-\frac{{\hat{k}}_{\mathrm{avg}}}{r+1}. \end{array}$$

□

**Remark 1.**  *Since a $\left(\hat{v},\hat{k},1\right)$-BIBD is also a $\left(\hat{v},\hat{k},1\right)$-PBD, our construction in Theorem 1 includes the code constructed in [15]. When H is an incidence matrix of a $\left(\hat{v},\hat{k},1\right)$-BIBD, the above proof process is still valid, and we obtain an $\left(n=\hat{b},k,r=\hat{r}-1,t=\hat{k}\right)$ LRC with all-symbol availability, where d(C)≥t+1 and $R\left(C\right)\geq 1-\frac{t}{r+1}$.*

*Note that in our construction in this subsection, the size of the parity-check matrix H may not be (n−k)×n. In a full-rank (n−k)×n parity-check matrix H, if we add several redundant rows to H, where the extra rows are all linear combinations of the first (n−k) rows, then the resulting parity-check matrix $H^{\prime }$ is still a parity-check matrix of the same code, provided that H and $H^{\prime }$ define the same linear space. In the above construction, it is relatively difficult to obtain the exact rank of H for arbitrary parameters, which will be the subject of future work. However, in the next section, we partially solve this problem.*

*This work generalizes the construction in [23] by assigning equal locality but varying availability. It is well suited for distributed storage, as it increases the number of repair sets for high-load or failure-prone nodes, thereby enhancing fault tolerance.*

We illustrate the code construction in the above theorem with some examples.

**Example 2.**  *Let M represent the incidence matrix of a (7,3,1)-BIBD. By removing a single point from this BIBD, a (6,{2,3},1)-PBD with points 1,2,3,4,5,6 and blocks {1,2,4},{1,3,5},{1,6},{2,3},{2,5,6},{3,4,6},{4,5} can be constructed. The incidence matrix of this PBD, denoted as H, acts as the parity-check matrix for an LRC:*

(6) $$M=\left(\begin{array}{ccccccc} 1 & 1 & 1 & 0 & 0 & 0 & 0\\ 1 & 0 & 0 & 1 & 1 & 0 & 0\\ 0 & 1 & 0 & 1 & 0 & 1 & 0\\ 1 & 0 & 0 & 0 & 0 & 1 & 1\\ 0 & 1 & 0 & 0 & 1 & 0 & 1\\ 0 & 0 & 1 & 0 & 1 & 1 & 0\\ 0 & 0 & 1 & 1 & 0 & 0 & 1 \end{array}\right),H=\left(\begin{array}{ccccccc} 1 & 1 & 1 & 0 & 0 & 0 & 0\\ 1 & 0 & 0 & 1 & 1 & 0 & 0\\ 0 & 1 & 0 & 1 & 0 & 1 & 0\\ 1 & 0 & 0 & 0 & 0 & 1 & 1\\ 0 & 1 & 0 & 0 & 1 & 0 & 1\\ 0 & 0 & 1 & 0 & 1 & 1 & 0 \end{array}\right).$$

*Since the row rank of H is 4, we obtain a (7,3,2,2)-LRC. This code maintains the same locality while providing nonuniform availabilities for different coordinates. The minimum distance of the code is *4*, and its rate is 3/7. Furthermore, this code achieves the upper bound on the distances given in (2) and (3), making it distance-optimal. Without loss of generality, assume that in a codeword c, $c_{2}$ is lost. There are three rows (rows *1*, *3*, and *5*) with nonzero elements in the second column of H, leading to the following three linear equations: $c_{1}+c_{2}+c_{3}=0,\;c_{2}+c_{4}+c_{6}=0,\;$ and $c_{2}+c_{5}+c_{7}=0$. Since our code is constructed over $F_{2}$, $c_{2}$ can be recovered by XORing $c_{1}$ and $c_{3}$, XORing $c_{4}$ and $c_{6}$, or XORing $c_{5}$ and $c_{7}$. The recovery sets of $c_{2}$ are $R_{1}\left(2\right)=\left\{1,3\right\},R_{2}\left(2\right)=\left\{4,6\right\},$ and $R_{3}\left(2\right)=\left\{5,7\right\}$.*

*Similarly, consider $M^{\prime }$, the incidence matrix of a (9,3,1)-BIBD. By deleting one point from this BIBD, an (8,{2,3},1)-PBD is obtained. The incidence matrix of this PBD, $H^{\prime }$, serves as the parity-check matrix for a (12,4,3,2)-LRC. This code also features the same locality and nonuniform availabilities. This example has a minimum distance of *5* and a rate of 1/3:*

(7) $$\begin{array}{c} M^{\prime }=\left(\begin{array}{cccccccccccc} 1 & 1 & 1 & 1 & 0 & 0 & 0 & 0 & 0 & 0 & 0 & 0\\ 1 & 0 & 0 & 0 & 1 & 1 & 1 & 0 & 0 & 0 & 0 & 0\\ 0 & 1 & 0 & 0 & 1 & 0 & 0 & 1 & 1 & 0 & 0 & 0\\ 0 & 0 & 1 & 0 & 1 & 0 & 0 & 0 & 0 & 1 & 1 & 0\\ 0 & 1 & 0 & 0 & 0 & 1 & 0 & 0 & 0 & 1 & 0 & 1\\ 1 & 0 & 0 & 0 & 0 & 0 & 0 & 1 & 0 & 0 & 1 & 1\\ 0 & 0 & 1 & 0 & 0 & 0 & 1 & 0 & 1 & 0 & 0 & 1\\ 0 & 0 & 0 & 1 & 0 & 1 & 0 & 0 & 1 & 0 & 1 & 0\\ 0 & 0 & 0 & 1 & 0 & 0 & 1 & 1 & 0 & 1 & 0 & 0 \end{array}\right),\\ H^{\prime }=\left(\begin{array}{cccccccccccc} 1 & 1 & 1 & 1 & 0 & 0 & 0 & 0 & 0 & 0 & 0 & 0\\ 1 & 0 & 0 & 0 & 1 & 1 & 1 & 0 & 0 & 0 & 0 & 0\\ 0 & 1 & 0 & 0 & 1 & 0 & 0 & 1 & 1 & 0 & 0 & 0\\ 0 & 0 & 1 & 0 & 1 & 0 & 0 & 0 & 0 & 1 & 1 & 0\\ 0 & 1 & 0 & 0 & 0 & 1 & 0 & 0 & 0 & 1 & 0 & 1\\ 1 & 0 & 0 & 0 & 0 & 0 & 0 & 1 & 0 & 0 & 1 & 1\\ 0 & 0 & 1 & 0 & 0 & 0 & 1 & 0 & 1 & 0 & 0 & 1\\ 0 & 0 & 0 & 1 & 0 & 1 & 0 & 0 & 1 & 0 & 1 & 0 \end{array}\right). \end{array}$$

In the above construction, the locality *r* of the code is fixed to $\hat{r}-1$. However, using PBDs from resolvable BIBDs, we can construct locally repairable codes with more choices for the locality *r*. First, choose (r+1) parallel classes from a $\left(\hat{v}-1,\hat{r},\left\{\hat{k},\hat{k}-1\right\},1\right)$-PBD constructed by removing a single point in a $\left(\hat{v},\hat{b},\hat{r},\hat{k},1\right)$-RBIBD. Each parallel class corresponds to a $\left(\hat{v}-1\right)\times \frac{\hat{v}}{\hat{k}}$ submatrix $M_{i}$. Choose r+1 such submatrices, where $\left(r+1\right)\frac{\hat{v}}{\hat{k}}\geq \hat{v}$, i.e., $\left(r+1\right)\geq \hat{k}$. Since there are only $\hat{r}$ parallel classes in the PBD, $r+1\leq \hat{r}$, we obtain a $\left(\hat{v}-1\right)\times \left(r+1\right)\frac{\hat{v}}{\hat{k}}$ matrix *H*:

(8) $$H=\left(\begin{array}{ccc} M_{1} & \ldots & M_{r+1} \end{array}\right).$$

Note that in *H*, the weight of each row is (r+1), and the weight of each column is $\hat{k}$ or $\hat{k}-1$.

**Theorem 2.**  *H defines an ${\left(n=\left(r+1\right)\frac{\hat{v}}{\hat{k}},k,r\in \left[t,\hat{r}-1\right],t=\hat{k}-1\right)}_{2}$ locally repairable code C. This code provides all-symbol (r,t)-locality, the minimum distance satisfies d(C)≥t+1, and the code rate is bounded below by $R\left(C\right)\geq 1-\frac{\left(r+1\right)}{\left(t+1\right)}\frac{\left(\hat{v}-1\right)}{\hat{v}}$.*

**Proof.**  Note that in our construction of *H*, any two rows intersect at most once. If two rows in *H* intersect twice, two points will appear twice in the related PBD. So, for each coordinate in the code, there are at least $t=\hat{k}-1$ repair sets of size *r*. We have shown that our construction provides all-symbol (r,t)-locality.

Assume that c is a randomly chosen nonzero codeword from C and *i* denotes the first nonzero element in C. Since each coordinate in C has *t* disjoint recovery sets, the minimum hamming weight of the codeword is t+1, where d(C)≥t+1. The code rate can be computed as follows:

(9) $$\begin{array}{cc} R(C) & =1-\frac{\mathrm{rank}(H)}{n}\\ \; & \geq 1-\frac{\left(\hat{v}-1\right)}{n}\\ \; & =1-\frac{\left(r+1\right)}{\left(t+1\right)}\frac{\left(\hat{v}-1\right)}{\hat{v}} \end{array}$$

□

In this construction, we constructed locally repairable codes with uniform locality $r\in [t,\hat{r}-1]$ and nonuniform availability. We provide some examples below to illustrate the above codes from our construction.

**Example 3.**  *$M_{1}$ is the incidence matrix of a (9,3,1)-RBIBD. By removing a point in this RBIBD, we obtain the incidence matrix $M_{2}$ of an (8,{2,3},1)-PBD:*

(10) $$M_{1}=\left(\begin{array}{cccccccccccc} 1 & 0 & 0 & 1 & 0 & 0 & 1 & 0 & 0 & 1 & 0 & 0\\ 1 & 0 & 0 & 0 & 1 & 0 & 0 & 1 & 0 & 0 & 1 & 0\\ 0 & 1 & 0 & 1 & 0 & 0 & 0 & 0 & 1 & 0 & 1 & 0\\ 0 & 0 & 1 & 0 & 0 & 1 & 1 & 0 & 0 & 0 & 1 & 0\\ 0 & 0 & 1 & 1 & 0 & 0 & 0 & 1 & 0 & 0 & 0 & 1\\ 1 & 0 & 0 & 0 & 0 & 1 & 0 & 0 & 1 & 0 & 0 & 1\\ 0 & 1 & 0 & 0 & 1 & 0 & 1 & 0 & 0 & 0 & 0 & 1\\ 0 & 1 & 0 & 0 & 0 & 1 & 0 & 1 & 0 & 1 & 0 & 0\\ 0 & 0 & 1 & 0 & 1 & 0 & 0 & 0 & 1 & 1 & 0 & 0 \end{array}\right),$$

(11) $$M_{2}=\left(\begin{array}{cccccccccccc} 1 & 0 & 0 & 0 & 1 & 0 & 0 & 1 & 0 & 0 & 1 & 0\\ 0 & 1 & 0 & 1 & 0 & 0 & 0 & 0 & 1 & 0 & 1 & 0\\ 0 & 0 & 1 & 0 & 0 & 1 & 1 & 0 & 0 & 0 & 1 & 0\\ 0 & 0 & 1 & 1 & 0 & 0 & 0 & 1 & 0 & 0 & 0 & 1\\ 1 & 0 & 0 & 0 & 0 & 1 & 0 & 0 & 1 & 0 & 0 & 1\\ 0 & 1 & 0 & 0 & 1 & 0 & 1 & 0 & 0 & 0 & 0 & 1\\ 0 & 1 & 0 & 0 & 0 & 1 & 0 & 1 & 0 & 1 & 0 & 0\\ 0 & 0 & 1 & 0 & 1 & 0 & 0 & 0 & 1 & 1 & 0 & 0 \end{array}\right),$$

*Choose the first three parallel classes in $M_{2}$ to form H:*

(12) $$H=\left(\begin{array}{ccccccccc} 1 & 0 & 0 & 0 & 1 & 0 & 0 & 1 & 0\\ 0 & 1 & 0 & 1 & 0 & 0 & 0 & 0 & 1\\ 0 & 0 & 1 & 0 & 0 & 1 & 1 & 0 & 0\\ 0 & 0 & 1 & 1 & 0 & 0 & 0 & 1 & 0\\ 1 & 0 & 0 & 0 & 0 & 1 & 0 & 0 & 1\\ 0 & 1 & 0 & 0 & 1 & 0 & 1 & 0 & 0\\ 0 & 1 & 0 & 0 & 0 & 1 & 0 & 1 & 0\\ 0 & 0 & 1 & 0 & 1 & 0 & 0 & 0 & 1 \end{array}\right),$$

*We obtain a (9,2,2,3) locally repairable code with uniform locality and nonuniform availability, with d(C)=6 and $R\left(C\right)=\frac{2}{9}$. The coordinates 2,3,5,7, and *8* have three disjoint repair sets of size *2*. Assume that in a codeword c, the symbol $c_{3}$ is erased. The three recovery sets of $c_{3}$ are $R_{1}\left(3\right)=\left\{6,7\right\},R_{2}\left(3\right)=\left\{4,8\right\},$ and $R_{3}\left(3\right)=\left\{5,9\right\}$. $c_{3}$ can be recovered by adding all symbols indexed by any repair set (for example, $c_{5}+c_{9}=c_{3}$). The code rate of the above construction is not sufficiently high. However, it can be used to construct an IS-LRC, and its transpose can be used to construct locally repairable codes with nonuniform localities.*

### 3.2. Locally Repairable Codes with Nonuniform Localities

In this subsection, we introduce a method for constructing locally repairable codes with varying localities but uniform availability, derived from PBDs. These constructions ensure all-symbol (r,t)-availability.

Consider a $\left(\hat{v},\hat{b},\hat{r},\hat{k},1\right)$-RBIBD, where $\hat{r}=\frac{\left(\hat{v}-1\right)}{\hat{k}-1}$ and $\hat{b}=\frac{\hat{v}\hat{r}}{\hat{k}}=\frac{\left({\hat{v}}^{2}-\hat{v}\right)}{{\hat{k}}^{2}-\hat{k}}$. Let the transpose of the incidence matrix of this RBIBD be denoted by *M*. This RBIBD contains $\hat{r}$ parallel classes. *M* contains $\hat{r}$ submatrices, denoted by $M_{1},\ldots ,M_{\hat{r}}$:

(13) $$M=\left(\begin{array}{c} M_{1}\\ \vdots \\ M_{\hat{r}} \end{array}\right).$$

Each submatrix $M_{i}$ corresponds to a parallel class in the RBIBD. Then, a $\left(\hat{v}-1,\left\{\hat{k},\hat{k}-1\right\},1\right)$-PBD can be obtained by removing a single point from the RBIBD. The transpose of the incidence matrix corresponding to this PBD, denoted by $M^{\prime }$, also contains $\hat{r}$ submatrices as follows:

(14) $$M^{\prime }=\left(\begin{array}{c} M_{1}^{\prime }\\ \vdots \\ M_{\hat{r}}^{\prime } \end{array}\right).$$

Here, $M^{\prime }$ is a $\hat{b}\times \left(\hat{v}-1\right)$ matrix. Next, choose any *t* submatrices $M_{1}^{\prime },\ldots ,M_{t}^{\prime }$ from the collection of submatrices $\left\{M_{1}^{\prime },\ldots ,M_{\hat{r}}^{\prime }\right\}$. Then, *H* is defined as follows:

(15) $$H=\left(\begin{array}{c} M_{1}^{\prime }\\ \vdots \\ M_{t}^{\prime } \end{array}\right).$$

In the above block design, $\hat{r}\geq \hat{k}+1$ is satisfied. Then, select $t\leq \hat{k}-1$ submatrices from *M*. Consequently, the resulting matrix *H* is a $\left(\hat{v}-1\right)\times t\frac{\hat{v}}{\hat{k}}$ matrix.

**Theorem 3.**  *The code constructed with H as its parity-check matrix is an ${\left(n=\hat{v}-1,k,r=\hat{k}-1,t\leq r\right)}_{2}$ locally repairable code. This code has varying localities but uniform availability while possessing all-symbol locality. In this construction, $R\left(C\right)\geq 1-\frac{t(n+1)}{(r+1)n}-\frac{t-1}{n}$, d(C)≥t+1.*

**Proof.**  By the construction of *H*, it is clear that *H* is formed by selecting *t* parallel classes from *M* and removing the same point from all blocks within these parallel classes. Additionally, *H* is part of the incidence matrix of the PBD. Therefore, any two different points appear in one block. When forming the PBD, one point is removed from the RBIBD, resulting in a PBD with block sizes $\hat{k}$ or $\hat{k}-1$. In this process, the frequency of occurrence of each remaining point does not change, and the $\hat{v}-1$ remaining points appear $\hat{r}$ times in $M^{\prime }$.

From this construction, we observe that each row of *H* has a weight of at most $\hat{k}=r+1$, while each column of *H* has a weight *t*. The column weight equals *t* because each point remaining in the RBIBD is present once in each submatrix of $M^{\prime }$, and *t* submatrices $M_{1}^{\prime },\ldots ,M_{t}^{\prime }$ are selected to construct *H*. Therefore, every symbol in the codeword has availability *t*. The locality of this symbol is *r*. Because blocks belonging to distinct parallel classes share exactly one point, the *t* recovery sets are mutually disjoint (If two recovery sets had more than one common point, it would indicate that two blocks from separate parallel classes intersect at two points, contradicting the fundamental properties of PBDs). Therefore, the code constructed in this theorem is an ${\left(n=\hat{v}-1,k,r=\hat{k}-1,t\leq r\right)}_{2}$ AS-LRC, d(C)≥t+1.

*H* has $m=t\frac{\hat{v}}{\hat{k}}$ rows and *n* columns. Note that *H* is not a full-rank matrix. Choose one parallel class in *H* and fix this parallel class. Note that each parallel class is a partition of *n*. So, for each of the remaining parallel classes in *H*, there exists a redundant row *R*, and this row can be represented by the fixed parallel class and the corresponding parallel class. So, we can remove t−1 redundant rows from *H*. Thus, the rank of *H* satisfies $\mathrm{rank}\left(H\right)\leq t\frac{\hat{v}}{\hat{k}}-\left(t-1\right)$. Let the rate of the code be denoted by R(C). Then, the rate is given by

$$\begin{array}{cc} R(C) & =1-\frac{\mathrm{rank}(H)}{n}\\ \; & \geq 1-\frac{t\frac{\hat{v}}{\hat{k}}-t+1}{n}\\ \; & =1-\frac{t(n+1)}{(r+1)n}-\frac{t-1}{n}. \end{array}$$

□

**Example 4.**  *Let M denote the transpose of the incidence matrix of a (9,3,1)-RBIBD. Construct a matrix $H_{1}$ by selecting two parallel classes from M. The matrix $H_{1}$ serves as the parity-check matrix for a ${\left(9,4,2,2\right)}_{2}$ SA-LRC C, which has a minimum distance of *4* and a rate of 4/9.*

*By removing one point (corresponding to the RBIBD) from $H_{1}$, a new matrix $H_{2}$ is obtained. The matrix $H_{2}$ acts as the parity-check matrix for an ${\left(8,3,2,2\right)}_{2}$ locally repairable code C with varying localities but uniform availability. This code achieves a minimum distance of *4* and a rate of 3/8.*

(16) $$M=\left(\begin{array}{ccccccccc} 1 & 1 & 0 & 0 & 0 & 1 & 0 & 0 & 0\\ 0 & 0 & 1 & 0 & 0 & 0 & 1 & 1 & 0\\ 0 & 0 & 0 & 1 & 1 & 0 & 0 & 0 & 1\\ 1 & 0 & 1 & 0 & 1 & 0 & 0 & 0 & 0\\ 0 & 1 & 0 & 0 & 0 & 0 & 1 & 0 & 1\\ 0 & 0 & 0 & 1 & 0 & 1 & 0 & 1 & 0\\ 1 & 0 & 0 & 1 & 0 & 0 & 1 & 0 & 0\\ 0 & 1 & 0 & 0 & 1 & 0 & 0 & 1 & 0\\ 0 & 0 & 1 & 0 & 0 & 1 & 0 & 0 & 1\\ 1 & 0 & 0 & 0 & 0 & 0 & 0 & 1 & 1\\ 0 & 1 & 1 & 1 & 0 & 0 & 0 & 0 & 0\\ 0 & 0 & 0 & 0 & 1 & 1 & 1 & 0 & 0 \end{array}\right).$$

(17) $$H_{1}=\left(\begin{array}{ccccccccc} 1 & 1 & 0 & 0 & 0 & 1 & 0 & 0 & 0\\ 0 & 0 & 1 & 0 & 0 & 0 & 1 & 1 & 0\\ 0 & 0 & 0 & 1 & 1 & 0 & 0 & 0 & 1\\ 1 & 0 & 1 & 0 & 1 & 0 & 0 & 0 & 0\\ 0 & 1 & 0 & 0 & 0 & 0 & 1 & 0 & 1\\ 0 & 0 & 0 & 1 & 0 & 1 & 0 & 1 & 0 \end{array}\right),H_{2}=\left(\begin{array}{cccccccc} 1 & 1 & 0 & 0 & 0 & 1 & 0 & 0\\ 0 & 0 & 1 & 0 & 0 & 0 & 1 & 1\\ 0 & 0 & 0 & 1 & 1 & 0 & 0 & 0\\ 1 & 0 & 1 & 0 & 1 & 0 & 0 & 0\\ 0 & 1 & 0 & 0 & 0 & 0 & 1 & 0\\ 0 & 0 & 0 & 1 & 0 & 1 & 0 & 1 \end{array}\right).$$

*It is important to note that while the code defined by $H_{2}$ has a lower rate compared to the code defined by $H_{1}$, the locality r of certain nodes (the coordinates 2,4,5,7) in the code corresponding to $H_{2}$ is reduced to *1*. This reduction in locality decreases the repair bandwidth for these nodes when node failure happens. Additionally, the relative distance of the code defined by $H_{2}$ is improved, enhancing the overall reliability of the code. Consider the case where $c_{4}$ is erased from the codeword c∈C. The two repair sets of $c_{4}$ are $R_{1}\left(4\right)=\left\{5\right\}$ and $R_{2}\left(4\right)=\left\{6,8\right\}$. So, $c_{4}$ can be recovered by accessing $c_{5}$ or XORing $c_{6}$ and $c_{8}$.*

**Remark 2.**  *We can choose t parallel classes from M (an incidence matrix of a $\left(\hat{v},\hat{b},\hat{r},\hat{k},1\right)$-RBIBD) and form H as described above. In this case, the above proof process is still applicable. We can obtain an $\left(n=\hat{v}.k,r=\hat{k}-1,t\leq \hat{k}\right)$ AS-LRC C with d(C)≥t+1 and $R\left(C\right)\geq 1-\frac{t}{\left(r+1\right)}-\frac{t-1}{n}$. Since an RBIBD is also a PBD, our construction in Theorem 3 includes the parameters of the code proposed in [15]. The first code in Example 4 is from this construction.*

### 3.3. Construction of Binary Locally Repairable Codes with Different Information-Symbol Availabilities

In Section 3.1, a construction of locally repairable codes with different availabilities and the same locality from PBDs was presented. In distributed storage systems, information symbols are typically accessed more frequently because they represent the original data, which can usually be accessed without decoding. Therefore, if a class of LRCs with different availabilities for information symbols can be constructed using PBDs, such codes would be more suitable for distributed storage systems than the codes presented in Section 3.1.

This subsection provides a construction of LRCs with different information-symbol availabilities. The construction is based on constructing the generator matrix *G* or parity-check matrix *H*:

(18) $$\begin{array}{c} G=\left(\begin{array}{cc} I_{k\times k}\;| & R^{T} \end{array}\right),\\ H=\left(\begin{array}{cc} -R\;| & I_{\left(n-k)\times (n-k\right)} \end{array}\right)\\ =\left(\begin{array}{cc} R\;| & I_{\left(n-k)\times (n-k\right)} \end{array}\right). \end{array}$$

The last equation follows from the fact that our codes are over $F_{2}$. Let (X,B) be a $\left(\hat{v},\hat{K},1\right)$-PBD, where each point appears exactly $\hat{r}$ times in B, and the total number of blocks is $\left|B\right|=\hat{b}$. Let *R* represent the incidence matrix of this PBD. Assume that for every $\hat{k}\in \hat{K}$, there exists at least one block of size $\hat{k}$. Let ${\hat{k}}_{\min}$, ${\hat{k}}_{\max}$, and ${\hat{k}}_{\mathrm{avg}}$ denote the smallest, largest, and average block sizes in B, respectively.

**Theorem 4.**  *The code constructed above is an ${\left(n=\hat{b}+\hat{v},k=\hat{b},r=\hat{r},t={\hat{k}}_{\min}\right)}_{2}$ locally repairable code. This code has uniform locality and nonuniform availability for all information symbols. It achieves a minimum distance of at least t+1 and has a rate of $R\left(C\right)=\frac{\hat{b}}{\hat{b}+\hat{v}}$. Additionally, the code provides all-symbol locality.*

**Proof.**  Since *R* acts as the parity-check matrix of an $\left(n^{\prime }=\hat{b},k^{\prime },r^{\prime }=\hat{r}-1,t^{\prime }={\hat{k}}_{\min}\right)$ locally repairable code, every symbol in *R* has availability $t^{\prime }$. Furthermore, any two rows of *R* intersect at exactly one point. Each row of *R* has a weight of $r=\hat{r}$, and each column of *R* has a weight of $t={\hat{k}}_{\min}$. This indicates that each message symbol in the code is safeguarded by at least *t* different recovery sets, where each set has a size of *r*. These recovery sets enable *t* independent and parallel repair processes for every symbol. Furthermore, some information symbols are secured by more than *t* disjoint recovery sets of size *r*. Consequently, the code constructed in this section is an $\left(n=\hat{b}+\hat{v},k=\hat{v},r=\hat{r},t={\hat{k}}_{\min}\right)$ LRC, featuring uniform locality for the information symbols and nonuniform availability.

We now proceed to examine the minimum distance of the code. Here, *H* represents the parity-check matrix:

(19) $$\begin{array}{cc} H & =\left(\begin{array}{cc} R\;| & I_{\hat{v}\times \hat{v}} \end{array}\right)\\ \; & =\left(\begin{array}{cc} R\;| & I_{\left(n-k)\times (n-k\right)} \end{array}\right). \end{array}$$

c is a codeword with weight *d*, where $c_{i}$ denotes the *i*th nonzero symbol of c. Assume that $c_{i}$ is located within the final $\hat{v}$ coordinates of c. In this scenario, at least one additional nonzero symbol $c_{j}$ must be present within the first *k* coordinates of c. *R* serves as the parity-check matrix for an AS-LRC $\left(n^{\prime }=\hat{b},k^{\prime },r^{\prime }=\hat{r}-1,t^{\prime }={\hat{k}}_{\min}\right)$; thus, it can be concluded that $c_{j}$ is involved in at least *t* parity-check equations. There exists a corresponding nonzero symbol $c_{h}$ in each of them. Moreover, the rows in *H* corresponding to these *t* equations satisfy the condition that the supports of different rows are mutually disjoint. Therefore, c must contain a minimum of t+1 nonzero elements.

Let us now examine the scenario where $c_{i}$ lies within the first *k* coordinates of c. In this case, $c_{i}$ is involved in no fewer than *t* parity-check equations. There is another nonzero symbol $c_{j}$ in each of these equations. Again, the supports of the corresponding rows in *H* are disjoint. Therefore, c contains at least t+1 nonzero entries. Thus, d(C)≥t+1. The rate of the constructed LRC is given by

(20) $$\begin{array}{c} R\left(C\right)=\frac{k}{n}=\frac{\hat{b}}{\hat{b}+\hat{v}}. \end{array}$$

□

**Remark 3.**  *Recall that a $\left(\hat{v},\hat{b},\hat{r},\hat{k},1\right)$-BIBD is also a $\left(\hat{v},\hat{b},\hat{r},\hat{k},1\right)$-PBD. R is its incidence matrix. Let $H=\left(\begin{array}{cc} R\;| & I_{\left(n-k)\times (n-k\right)} \end{array}\right)$. Then, the code C with H as its parity-check matrix is an $\left(n=\hat{v}+\hat{b},k=\hat{b},r=\hat{r},t=\hat{k}\right)$ IS-LRC with distance d(C)=t+1 and $R\left(C\right)=\frac{\hat{b}}{\hat{b}+\hat{v}}$. In this case, $\hat{b}\hat{k}=\hat{v}\hat{r}$ and $\frac{kt}{r}=\hat{v}=n-k$, so $R\left(C\right)=\frac{k}{n}=\frac{\hat{b}}{\hat{b}+\hat{v}}=\frac{r}{r+t}$, and d(C) is optimal. This construction includes a construction from [15].*

*We can also choose R as the transpose of the incidence matrix of a $\left(\hat{v},\hat{b},\hat{r},\hat{k},1\right)$-BIBD. Then, we can obtain an $\left(n=\hat{v}+\hat{b},k=\hat{v},r=\hat{k},t=\hat{r}\right)$ IS-LRC $C^{\prime }$ with distance d(C)=t+1 and $R\left(C\right)=\frac{\hat{v}}{\hat{b}+\hat{v}}$. Similarly, $R\left(C\right)=\frac{r}{r+t}$ and d(C) attain the bound in (3). This construction includes another code constructed in [15].*

**Example 5.**  *Let M represent the incidence matrix of a (7,3,1)-BIBD. By applying the construction method described in Theorem 1, removing a single point from this BIBD produces a (6,{2,3},1)-PBD. The resulting incidence matrix H serves as the parity-check matrix for a (7,3,2,2)-LRC with all-symbol locality and nonuniform availability. This code has a minimum distance of *4* and a rate of 3/7. Using the construction outlined in this section, a generator matrix G for a (13,7,3,2)-LRC C can be derived from H. In this code, some information symbols (coordinates 1,2,5,6) are protected by three disjoint recovery sets, making it an LRC with nonuniform availability for different information symbols. Its minimum distance is *3*, and its rate is 7/13. Assume that $c_{2}$ in a codeword c is erased. Its repair sets are $R_{1}\left(1\right)=\left\{2,3,8\right\},R_{2}\left(1\right)=\left\{4,5,9\right\},$ and $R_{3}\left(1\right)=\left\{6,7,11\right\}$. $c_{1}$ can be recovered by reading all symbols indexed by any of its recovery sets from c.*

(21) $$M=\left(\begin{array}{ccccccc} 1 & 1 & 1 & 0 & 0 & 0 & 0\\ 1 & 0 & 0 & 1 & 1 & 0 & 0\\ 0 & 1 & 0 & 1 & 0 & 1 & 0\\ 1 & 0 & 0 & 0 & 0 & 1 & 1\\ 0 & 1 & 0 & 0 & 1 & 0 & 1\\ 0 & 0 & 1 & 0 & 1 & 1 & 0\\ 0 & 0 & 1 & 1 & 0 & 0 & 1 \end{array}\right),H=\left(\begin{array}{ccccccc} 1 & 1 & 1 & 0 & 0 & 0 & 0\\ 1 & 0 & 0 & 1 & 1 & 0 & 0\\ 0 & 1 & 0 & 1 & 0 & 1 & 0\\ 1 & 0 & 0 & 0 & 0 & 1 & 1\\ 0 & 1 & 0 & 0 & 1 & 0 & 1\\ 0 & 0 & 1 & 0 & 1 & 1 & 0 \end{array}\right),$$

(22) $$G=\left(\begin{array}{ccccccccccccc} 1 & 0 & 0 & 0 & 0 & 0 & 0 & 1 & 1 & 0 & 1 & 0 & 0\\ 0 & 1 & 0 & 0 & 0 & 0 & 0 & 1 & 0 & 1 & 0 & 1 & 0\\ 0 & 0 & 1 & 0 & 0 & 0 & 0 & 1 & 0 & 0 & 0 & 0 & 1\\ 0 & 0 & 0 & 1 & 0 & 0 & 0 & 0 & 1 & 1 & 0 & 0 & 0\\ 0 & 0 & 0 & 0 & 1 & 0 & 0 & 0 & 1 & 0 & 0 & 1 & 1\\ 0 & 0 & 0 & 0 & 0 & 1 & 0 & 0 & 0 & 1 & 1 & 0 & 1\\ 0 & 0 & 0 & 0 & 0 & 0 & 1 & 0 & 0 & 0 & 1 & 1 & 0 \end{array}\right).$$

*Similarly, let $M^{\prime }$ denote the incidence matrix of a (9,3,1)-BIBD. Removing one point from this BIBD results in an (8,{2,3},1)-PBD whose incidence matrix is $H^{\prime }$. $H^{\prime }$ is the parity-check matrix of a (12,4,3,2)-LRC, which also has all-symbol locality and nonuniform availabilities. This code has a minimum distance of *5* and a rate of 1/3. Using the construction presented in this section, a generator matrix $G^{\prime }$ for a (20,12,4,2)-LRC $C^{\prime }$ can be obtained from $H^{\prime }$. In this code, some information symbols have three disjoint recovery sets, making it an LRC with varying information-symbol availabilities. Its minimum distance is *3*, and its rate is 3/5. Consider a message symbol $c_{3}$ from a codeword $c\in C^{\prime }$ that cannot be accessed. The recovery sets of $c_{3}$ are $R_{1}\left(3\right)=\left\{1,2,13\right\},R_{2}\left(3\right)=\left\{5,10,11,16\right\},$ and $R_{3}\left(3\right)=\left\{7,9,12,19\right\}$, and each repair set contains only one parity symbol. $c_{3}$ can be reconstructed by XORing all symbols in any of its repair sets.*

(23) $$M^{\prime }=\left(\begin{array}{cccccccccccc} 1 & 1 & 1 & 1 & 0 & 0 & 0 & 0 & 0 & 0 & 0 & 0\\ 1 & 0 & 0 & 0 & 1 & 1 & 1 & 0 & 0 & 0 & 0 & 0\\ 0 & 1 & 0 & 0 & 1 & 0 & 0 & 1 & 1 & 0 & 0 & 0\\ 0 & 0 & 1 & 0 & 1 & 0 & 0 & 0 & 0 & 1 & 1 & 0\\ 0 & 1 & 0 & 0 & 0 & 1 & 0 & 0 & 0 & 1 & 0 & 1\\ 1 & 0 & 0 & 0 & 0 & 0 & 0 & 1 & 0 & 0 & 1 & 1\\ 0 & 0 & 1 & 0 & 0 & 0 & 1 & 0 & 1 & 0 & 0 & 1\\ 0 & 0 & 0 & 1 & 0 & 1 & 0 & 0 & 1 & 0 & 1 & 0\\ 0 & 0 & 0 & 1 & 0 & 0 & 1 & 1 & 0 & 1 & 0 & 0 \end{array}\right),H^{\prime }=\left(\begin{array}{cccccccccccc} 1 & 1 & 1 & 1 & 0 & 0 & 0 & 0 & 0 & 0 & 0 & 0\\ 1 & 0 & 0 & 0 & 1 & 1 & 1 & 0 & 0 & 0 & 0 & 0\\ 0 & 1 & 0 & 0 & 1 & 0 & 0 & 1 & 1 & 0 & 0 & 0\\ 0 & 0 & 1 & 0 & 1 & 0 & 0 & 0 & 0 & 1 & 1 & 0\\ 0 & 1 & 0 & 0 & 0 & 1 & 0 & 0 & 0 & 1 & 0 & 1\\ 1 & 0 & 0 & 0 & 0 & 0 & 0 & 1 & 0 & 0 & 1 & 1\\ 0 & 0 & 1 & 0 & 0 & 0 & 1 & 0 & 1 & 0 & 0 & 1\\ 0 & 0 & 0 & 1 & 0 & 1 & 0 & 0 & 1 & 0 & 1 & 0 \end{array}\right)$$

(24) $$G^{\prime }=\left(\begin{array}{cccccccccccccccccccc} 1 & 0 & 0 & 0 & 0 & 0 & 0 & 0 & 0 & 0 & 0 & 0 & 1 & 1 & 0 & 0 & 0 & 1 & 0 & 0\\ 0 & 1 & 0 & 0 & 0 & 0 & 0 & 0 & 0 & 0 & 0 & 0 & 1 & 0 & 1 & 0 & 1 & 0 & 0 & 0\\ 0 & 0 & 1 & 0 & 0 & 0 & 0 & 0 & 0 & 0 & 0 & 0 & 1 & 0 & 0 & 1 & 0 & 0 & 1 & 0\\ 0 & 0 & 0 & 1 & 0 & 0 & 0 & 0 & 0 & 0 & 0 & 0 & 1 & 0 & 0 & 0 & 0 & 0 & 0 & 1\\ 0 & 0 & 0 & 0 & 1 & 0 & 0 & 0 & 0 & 0 & 0 & 0 & 0 & 1 & 1 & 1 & 0 & 0 & 0 & 0\\ 0 & 0 & 0 & 0 & 0 & 1 & 0 & 0 & 0 & 0 & 0 & 0 & 0 & 1 & 0 & 0 & 1 & 0 & 0 & 1\\ 0 & 0 & 0 & 0 & 0 & 0 & 1 & 0 & 0 & 0 & 0 & 0 & 0 & 1 & 0 & 0 & 0 & 0 & 1 & 0\\ 0 & 0 & 0 & 0 & 0 & 0 & 0 & 1 & 0 & 0 & 0 & 0 & 0 & 0 & 1 & 0 & 0 & 1 & 0 & 0\\ 0 & 0 & 0 & 0 & 0 & 0 & 0 & 0 & 1 & 0 & 0 & 0 & 0 & 0 & 1 & 0 & 0 & 0 & 1 & 1\\ 0 & 0 & 0 & 0 & 0 & 0 & 0 & 0 & 0 & 1 & 0 & 0 & 0 & 0 & 0 & 1 & 1 & 0 & 0 & 0\\ 0 & 0 & 0 & 0 & 0 & 0 & 0 & 0 & 0 & 0 & 1 & 0 & 0 & 0 & 0 & 1 & 0 & 1 & 0 & 1\\ 0 & 0 & 0 & 0 & 0 & 0 & 0 & 0 & 0 & 0 & 0 & 1 & 0 & 0 & 0 & 0 & 1 & 1 & 1 & 0 \end{array}\right).$$

As demonstrated in the examples above, the construction method presented in this section improves the rate of the codes described in Section 3.1. In this framework, the information symbols possess availability, whereas the parity symbols, despite lacking multiple disjoint recovery sets, still maintain locality *r*. Although this construction significantly enhances the performance of the codes compared to those in Section 3.1, it has certain limitations. The primary drawback is that the minimum distance of the construction is not optimal. The construction introduced in the next subsection aims to address this limitation.

### 3.4. Distance-Optimal Binary Locally Repairable Codes with Different Localities for Information Symbols

This section focuses on constructing a class of distance-optimal locally repairable codes with different localities while maintaining the same availability for information symbols. The design relies on the construction presented in Section 3.2:

(25) $$G=\left(\begin{array}{cc} I_{k\times k}\;| & R^{T} \end{array}\right),$$

(26) $$H=\left(\begin{array}{cc} R\;| & I_{\left(n-k)\times (n-k\right)} \end{array}\right).$$

*G* and *H* represent the generator matrix and parity-check matrix of the code, respectively. $M^{T}$ denotes the incidence matrix of a $\left(\hat{v},\hat{b},\hat{r},\hat{k},1\right)$-RBIBD. The matrix *M* contains $\hat{r}$ submatrices $\left\{M_{1},M_{2},\ldots ,M_{\hat{r}}\right\}$:

(27) $$M=\left(\begin{array}{c} M_{1}\\ \vdots \\ M_{\hat{r}} \end{array}\right).$$

Each submatrix $M_{i}$ corresponds to a parallel class in the RBIBD. By removing $s\leq \hat{k}-1$ points from the design, we obtain a matrix $M^{\prime }$, which can be partitioned into $\hat{r}$ submatrices:

(28) $$M^{\prime }=\left(\begin{array}{c} M_{1}^{\prime }\\ \vdots \\ M_{\hat{r}}^{\prime } \end{array}\right).$$

Note that in this case, $M^{\prime }$ is not necessarily an incidence matrix of a PBD. Here, $M^{\prime }$ has $\hat{b}$ rows and $\hat{v}-s$ columns. From submatrices $\left\{M_{1}^{\prime },M_{2}^{\prime },\ldots ,M_{\hat{r}}^{\prime }\right\}$, select any *t* submatrices $M_{1}^{\prime },\ldots ,M_{t}^{\prime }$. *R* is constructed as follows:

(29) $$R=\left(\begin{array}{c} M_{1}^{\prime }\\ \vdots \\ M_{t}^{\prime } \end{array}\right).$$

There are a total of $t<\frac{\hat{k}}{s}$ submatrices in *R*. The resulting matrix *R* has size $t\frac{\hat{v}}{\hat{k}}\times \left(\hat{v}-s\right)$, where $n-k=t\frac{\hat{v}}{\hat{k}}$. The weight of each row in *R* is at most $\hat{k}$, and the weight of each column in *R* is *t*.

In order to prove that the construction in this subsection is optimal, a lemma is required. The proof of this lemma is given below.

**Lemma 1**  ([15])**.** *Choose t parallel classes from the matrix M to form $R^{\prime }$. $H^{\prime }=\left(\begin{array}{cc} R^{\prime }\;| & I_{\left(n-k)\times (n-k\right)} \end{array}\right)$. Then, H is a parity-check matrix of an ${\left(n=\hat{v}+t\frac{\hat{v}}{\hat{k}},k=\hat{v},r=\hat{k},t<r\right)}_{2}$ IS-LRC $C^{\prime }$. The minimum distance is $d(C^{\prime })=t+1$, $C^{\prime }$ attains the bound in (3), and the rate of the code $R\left(C^{\prime }\right)=\frac{r}{r+t}$ attains the bound in (4) for t=2.*

**Proof.**  From our construction of $H^{\prime }$, we can see that the first *k* coordinates of $C^{\prime }$ are protected by *t* disjoint repair sets of size *r*. So, the code $C^{\prime }$ has information-symbol (r,t)-availability.

Next, we prove that this construction is distance-optimal. Recall that each information symbol has *t* disjoint recovery sets of size *r*. Choose any nonzero codeword c from $C^{\prime }$. Assume there is a nonzero symbol in c that is located in the *i*-th coordinate, and assume i∈[1,k]. Since each message symbol has *t* disjoint repair sets, there are at least *t* other nonzero coordinates in c. Now, consider the case when i∈[n−k,n]. From our construction of $H^{\prime }$, the last n−k columns of *H* are an identity matrix, so there is at least one nonzero coordinate in the first *k* coordinates. So, the Hamming weight of any codeword $c\in C^{\prime }$ is at least t+1.

Note that in this construction, every repair set contains exactly one parity symbol. Therefore,

(30) $$\begin{array}{cc} d(C) & \leq n-k-\left\lceil \frac{kt}{r}\right\rceil +t+1\\ \; & =\hat{v}+t\frac{\hat{v}}{\hat{k}}-\hat{v}-\left\lceil \frac{\hat{v}t}{\hat{k}}\right\rceil +t+1\\ \; & =t+1. \end{array}$$

So, this construction is distance-optimal, and $d(C^{\prime })=t+1$. The code rate $R\left(C^{\prime }\right)=\frac{k}{n}=\frac{\hat{v}}{\hat{v}+t\frac{\hat{v}}{\hat{k}}}=\frac{r}{r+t}$. □

**Theorem 5.**  *A code C with G as its generator matrix and H as its parity-check matrix has parameters ${\left(n=\hat{v}-s+t\frac{\hat{v}}{\hat{k}},k=\hat{v}-s,s+1\leq r=\hat{k},t<\frac{r}{s}\right)}_{2}$. This construction has nonuniform localities and uniform availability for all information symbols. Its minimum distance is t+1, and its rate is $R\left(C\right)=\frac{\hat{v}-s}{\hat{v}-s+t\frac{\hat{v}}{\hat{k}}}$. The minimum distance achieves the optimal bound stated in (3), and the code supports all-symbol locality.*

**Proof.**  (31) $$H=\left(\begin{array}{cc} R & |I_{t\frac{\hat{v}}{\hat{k}}\times t\frac{\hat{v}}{\hat{k}}} \end{array}\right)=\left(\begin{array}{cc} R & |I_{\left(n-k)\times (n-k\right)} \end{array}\right).$$

Here, $n-k=t\frac{\hat{v}}{\hat{k}},\;n=\hat{v}-s+t\frac{\hat{v}}{\hat{k}},$ and $\;k=\hat{v}-s$. From our construction of *H*, we can conclude that in this case, any two rows in *H* intersect at precisely one point in the first *k* columns of *H*. Therefore, every message symbol in the code can be recovered from *t* mutually disjoint repair sets, where each set contains at most $r=\hat{k}$ elements. This guarantees that each information coordinate can be retrieved in *t* different methods in parallel with repair sets of size at most *r*. Consequently, the code constructed in this theorem is an ${\left(n,k,r,t\right)}_{2}$ IS-LRC.

Then, we calculate the code’s minimum distance. Let c represent a nonzero codeword. Denote the *i*th nonzero symbol in c as $c_{i}$. First, suppose $c_{i}$ is one of the last $n-k=t\frac{\hat{v}}{\hat{k}}$ positions in c. From *H*, we know that at least one additional nonzero symbol $c_{j}$ is among the initial *k* coordinates of c. So, $c_{j}$ appears in at least *t* parity-check equations. Each of them involves at least one other nonzero symbol $c_{h}$, and the corresponding supports of rows in *H* are disjoint. Consequently, c contains at least t+1 nonzero symbols. Now, consider the case when $c_{i}$ comes from the first *k* coordinates of c. Since $c_{i}$ is involved in at least *t* parity-check equations, each of them involves another nonzero symbol $c_{j}$. Again, the rows in *H* corresponding to these equations are disjoint, so c contains at least t+1 nonzero elements. Therefore, the code’s minimum distance *d* satisfies d(C)≥t+1.

(32) $$R\left(C\right)=\frac{k}{n}=\frac{\hat{v}-s}{\hat{v}-s+t\frac{\hat{v}}{\hat{k}}}.$$

From Lemma 1, we know that an $\left(n^{\prime \prime }=n+s,k^{\prime \prime }=k+s,r^{\prime \prime }=r,t^{\prime \prime }=t\right)$ LRC $C^{\prime }$ with parity-check matrix $H^{\prime }$ is distance-optimal $d\left(C^{\prime }\right)=t^{\prime \prime }+1$. From the parameters of the RBIBD, we know that

(33) $$d\left(C^{\prime }\right)=n^{\prime \prime }-k^{\prime \prime }-\left\lceil \frac{k^{\prime \prime }t^{\prime \prime }}{r^{\prime \prime }}\right\rceil +t^{\prime \prime }+1=t^{\prime \prime }+1=t+1.$$

$d(C^{\prime })$ attains the bound given in (3). For LRCs where each repair set contains precisely one parity symbol [8],

(34) $$d\leq n-k-\left\lceil \frac{kt}{r}\right\rceil +t+1.$$

In the above construction, every recovery set has precisely one parity symbol. If d(C) satisfies this equality, the code is considered distance-optimal.

(35) $$\begin{array}{cc} d(C) & \leq n-k-\left\lceil \frac{kt}{r}\right\rceil +t+1\\ \; & =n^{\prime \prime }-k^{\prime \prime }-\left\lceil \frac{kt}{r}\right\rceil +t^{\prime \prime }+1 \end{array}$$

Now, we compare $\left\lceil \frac{kt}{r}\right\rceil$ and $\left\lceil \frac{k^{\prime \prime }t^{\prime \prime }}{r^{\prime \prime }}\right\rceil$. Note that $\frac{kt}{r}<\frac{k^{\prime \prime }t^{\prime \prime }}{r^{\prime \prime }}$. Since $\frac{k^{\prime \prime }t^{\prime \prime }}{r^{\prime \prime }}$ is an integer, if $\frac{k^{\prime \prime }t^{\prime \prime }}{r^{\prime \prime }}-\frac{kt}{r}<1$, $\left\lceil \frac{kt}{r}\right\rceil =\left\lceil \frac{k^{\prime \prime }t^{\prime \prime }}{r^{\prime \prime }}\right\rceil$.

(36) $$\begin{array}{cc} \; & \frac{k^{\prime \prime }t^{\prime \prime }}{r^{\prime \prime }}-\frac{kt}{r}\\ \; & =\frac{(k+s)t}{r}-\frac{kt}{r}\\ \; & =\frac{st}{r} \end{array}$$

In our construction, $t<\frac{r}{s}$, so $\frac{k^{\prime \prime }t^{\prime \prime }}{r^{\prime \prime }}-\frac{kt}{r}<1$. Then,

(37) $$\begin{array}{cc} d(C) & \leq n-k-\left\lceil \frac{kt}{r}\right\rceil +t+1\\ \; & =n^{\prime \prime }-k^{\prime \prime }-\left\lceil \frac{kt}{r}\right\rceil +t^{\prime \prime }+1\\ \; & =n^{\prime \prime }-k^{\prime \prime }-\left\lceil \frac{k^{\prime \prime }t^{\prime \prime }}{r^{\prime \prime }}\right\rceil +t^{\prime \prime }+1\\ \; & =t^{\prime \prime }+1\\ \; & =t+1. \end{array}$$

Combining this with the earlier result d(C)≥t+1, we conclude that d(C)=t+1, which attains the bound in (3). Hence, the LRC constructed in this theorem is distance-optimal. The proof is complete. □

**Remark 4.**  *When the chosen RBIBD in the above construction is an affine plane of order q, i.e., a $\left(\hat{v}=q^{2},\hat{b}=q\left(q+1\right),\hat{r}=q+1,\hat{k}=q,1\right)$-RBIBD, the parameters of the above construction can be written in a clearer form. The above construction yields an ${\left(n=r^{2}-s+tr,k=r^{2}-s,s+1\leq r=\hat{k},t<\frac{r}{s}\right)}_{2}$ distance-optimal LRC, with rate $R\left(C\right)=\frac{r^{2}-s}{r^{2}-s+tr}$.*

*Note that an RBIBD is a special case of a PBD. If we choose t submatrices from the above M, we can obtain a distance-optimal $\left(n=\hat{v}+t\frac{\hat{v}}{k},k=\hat{v},r=\hat{k},t\leq \hat{r}\right)$ IS-LRC C, where d(C)=t+1 and $R\left(C\right)=\frac{r}{r+t}$. These parameters include a construction from [15].*

**Example 6.**  *Let M be a matrix that is created by choosing two parallel classes from a (16,4,1)-RBIBD. By removing the final column of M, we derive a submatrix H, which corresponds to a portion of the incidence matrix of a (15,{4,3},1)-PBD. The matrix H serves as the parity-check matrix for a (15,8,3,2) locally repairable code (LRC) with nonuniform localities but uniform availability. In this code, certain symbols exhibit a locality of *2*. The minimum distance of the code is *4*, while its rate is 8/15. Note that this code attains the bound in (3), and its rate outperforms the upper bound on the rate in (4) for t=2.*

(38) $$M=\left(\begin{array}{cccccccccccccccc} 1 & 1 & 0 & 0 & 0 & 0 & 0 & 0 & 0 & 0 & 0 & 0 & 1 & 0 & 1 & 0\\ 0 & 0 & 1 & 0 & 1 & 1 & 1 & 0 & 0 & 0 & 0 & 0 & 0 & 0 & 0 & 0\\ 0 & 0 & 0 & 1 & 0 & 0 & 0 & 0 & 1 & 0 & 0 & 0 & 0 & 1 & 0 & 1\\ 0 & 0 & 0 & 0 & 0 & 0 & 0 & 1 & 0 & 1 & 1 & 1 & 0 & 0 & 0 & 0\\ 1 & 0 & 1 & 0 & 0 & 0 & 0 & 0 & 1 & 1 & 0 & 0 & 0 & 0 & 0 & 0\\ 0 & 1 & 0 & 0 & 0 & 0 & 1 & 0 & 0 & 0 & 0 & 1 & 0 & 0 & 0 & 1\\ 0 & 0 & 0 & 1 & 1 & 0 & 0 & 0 & 0 & 0 & 1 & 0 & 1 & 0 & 0 & 0\\ 0 & 0 & 0 & 0 & 0 & 1 & 0 & 1 & 0 & 0 & 0 & 0 & 0 & 1 & 1 & 0 \end{array}\right),$$

(39) $$H=\left(\begin{array}{ccccccccccccccc} 1 & 1 & 0 & 0 & 0 & 0 & 0 & 0 & 0 & 0 & 0 & 0 & 1 & 0 & 1\\ 0 & 0 & 1 & 0 & 1 & 1 & 1 & 0 & 0 & 0 & 0 & 0 & 0 & 0 & 0\\ 0 & 0 & 0 & 1 & 0 & 0 & 0 & 0 & 1 & 0 & 0 & 0 & 0 & 1 & 0\\ 0 & 0 & 0 & 0 & 0 & 0 & 0 & 1 & 0 & 1 & 1 & 1 & 0 & 0 & 0\\ 1 & 0 & 1 & 0 & 0 & 0 & 0 & 0 & 1 & 1 & 0 & 0 & 0 & 0 & 0\\ 0 & 1 & 0 & 0 & 0 & 0 & 1 & 0 & 0 & 0 & 0 & 1 & 0 & 0 & 0\\ 0 & 0 & 0 & 1 & 1 & 0 & 0 & 0 & 0 & 0 & 1 & 0 & 1 & 0 & 0\\ 0 & 0 & 0 & 0 & 0 & 1 & 0 & 1 & 0 & 0 & 0 & 0 & 0 & 1 & 1 \end{array}\right).$$

*Utilizing the construction outlined in this section, one can derive a generator matrix G for a (23,15,4,2) LRC C from H. This resulting code has a rate of 15/23, a minimum distance of *3*, and satisfies the distance-optimality bound. The construction presented here enhances the rate of the codes developed in Section 3.2 while maintaining distance optimality, making it particularly well suited for distributed storage applications. Suppose that an information symbol $c_{4}$ in a codeword c∈C is lost. The two repair sets of $c_{4}$ are $R_{1}\left(4\right)=\left\{9,14,18\right\}$ and $R_{2}\left(4\right)=\left\{5,11,13,22\right\}$, where each recovery set contains exactly one parity symbol. $c_{4}$ can be recovered by accessing all symbols indexed by $R_{1}\left(4\right)$ or $R_{2}\left(4\right)$ in c.*

(40) $$G=\left(\begin{array}{ccccccccccccccccccccccc} 1 & 0 & 0 & 0 & 0 & 0 & 0 & 0 & 0 & 0 & 0 & 0 & 0 & 0 & 0 & 1 & 0 & 0 & 0 & 1 & 0 & 0 & 0\\ 0 & 1 & 0 & 0 & 0 & 0 & 0 & 0 & 0 & 0 & 0 & 0 & 0 & 0 & 0 & 1 & 0 & 0 & 0 & 0 & 1 & 0 & 0\\ 0 & 0 & 1 & 0 & 0 & 0 & 0 & 0 & 0 & 0 & 0 & 0 & 0 & 0 & 0 & 0 & 1 & 0 & 0 & 1 & 0 & 0 & 0\\ 0 & 0 & 0 & 1 & 0 & 0 & 0 & 0 & 0 & 0 & 0 & 0 & 0 & 0 & 0 & 0 & 0 & 1 & 0 & 0 & 0 & 1 & 0\\ 0 & 0 & 0 & 0 & 1 & 0 & 0 & 0 & 0 & 0 & 0 & 0 & 0 & 0 & 0 & 0 & 1 & 0 & 0 & 0 & 0 & 1 & 0\\ 0 & 0 & 0 & 0 & 0 & 1 & 0 & 0 & 0 & 0 & 0 & 0 & 0 & 0 & 0 & 0 & 1 & 0 & 0 & 0 & 0 & 0 & 1\\ 0 & 0 & 0 & 0 & 0 & 0 & 1 & 0 & 0 & 0 & 0 & 0 & 0 & 0 & 0 & 0 & 1 & 0 & 0 & 0 & 1 & 0 & 0\\ 0 & 0 & 0 & 0 & 0 & 0 & 0 & 1 & 0 & 0 & 0 & 0 & 0 & 0 & 0 & 0 & 0 & 0 & 1 & 0 & 0 & 0 & 1\\ 0 & 0 & 0 & 0 & 0 & 0 & 0 & 0 & 1 & 0 & 0 & 0 & 0 & 0 & 0 & 0 & 0 & 1 & 0 & 1 & 0 & 0 & 0\\ 0 & 0 & 0 & 0 & 0 & 0 & 0 & 0 & 0 & 1 & 0 & 0 & 0 & 0 & 0 & 0 & 0 & 0 & 1 & 1 & 0 & 0 & 0\\ 0 & 0 & 0 & 0 & 0 & 0 & 0 & 0 & 0 & 0 & 1 & 0 & 0 & 0 & 0 & 0 & 0 & 0 & 1 & 0 & 0 & 1 & 0\\ 0 & 0 & 0 & 0 & 0 & 0 & 0 & 0 & 0 & 0 & 0 & 1 & 0 & 0 & 0 & 0 & 0 & 0 & 1 & 0 & 1 & 0 & 0\\ 0 & 0 & 0 & 0 & 0 & 0 & 0 & 0 & 0 & 0 & 0 & 0 & 1 & 0 & 0 & 1 & 0 & 0 & 0 & 0 & 0 & 1 & 0\\ 0 & 0 & 0 & 0 & 0 & 0 & 0 & 0 & 0 & 0 & 0 & 0 & 0 & 1 & 0 & 0 & 0 & 1 & 0 & 0 & 0 & 0 & 1\\ 0 & 0 & 0 & 0 & 0 & 0 & 0 & 0 & 0 & 0 & 0 & 0 & 0 & 0 & 1 & 1 & 0 & 0 & 0 & 0 & 0 & 0 & 1 \end{array}\right).$$

**Example 7.**  *We choose two parallel classes from a (25,5,1)-RBIBD to form $M^{\prime }$.*

(41) $$M^{\prime }=\left(\begin{array}{ccccccccccccccccccccccccc} 1 & 1 & 1 & 1 & 1 & 0 & 0 & 0 & 0 & 0 & 0 & 0 & 0 & 0 & 0 & 0 & 0 & 0 & 0 & 0 & 0 & 0 & 0 & 0 & 0\\ 0 & 0 & 0 & 0 & 0 & 1 & 1 & 1 & 1 & 1 & 0 & 0 & 0 & 0 & 0 & 0 & 0 & 0 & 0 & 0 & 0 & 0 & 0 & 0 & 0\\ 0 & 0 & 0 & 0 & 0 & 0 & 0 & 0 & 0 & 0 & 1 & 1 & 1 & 1 & 1 & 0 & 0 & 0 & 0 & 0 & 0 & 0 & 0 & 0 & 0\\ 0 & 0 & 0 & 0 & 0 & 0 & 0 & 0 & 0 & 0 & 0 & 0 & 0 & 0 & 0 & 1 & 1 & 1 & 1 & 1 & 0 & 0 & 0 & 0 & 0\\ 0 & 0 & 0 & 0 & 0 & 0 & 0 & 0 & 0 & 0 & 0 & 0 & 0 & 0 & 0 & 0 & 0 & 0 & 0 & 0 & 1 & 1 & 1 & 1 & 1\\ 1 & 0 & 0 & 0 & 0 & 1 & 0 & 0 & 0 & 0 & 1 & 0 & 0 & 0 & 0 & 1 & 0 & 0 & 0 & 0 & 1 & 0 & 0 & 0 & 0\\ 0 & 1 & 0 & 0 & 0 & 0 & 1 & 0 & 0 & 0 & 0 & 1 & 0 & 0 & 0 & 0 & 1 & 0 & 0 & 0 & 0 & 1 & 0 & 0 & 0\\ 0 & 0 & 1 & 0 & 0 & 0 & 0 & 1 & 0 & 0 & 0 & 0 & 1 & 0 & 0 & 0 & 0 & 1 & 0 & 0 & 0 & 0 & 1 & 0 & 0\\ 0 & 0 & 0 & 1 & 0 & 0 & 0 & 0 & 1 & 0 & 0 & 0 & 0 & 1 & 0 & 0 & 0 & 0 & 1 & 0 & 0 & 0 & 0 & 1 & 0\\ 0 & 0 & 0 & 0 & 1 & 0 & 0 & 0 & 0 & 1 & 0 & 0 & 0 & 0 & 1 & 0 & 0 & 0 & 0 & 1 & 0 & 0 & 0 & 0 & 1 \end{array}\right).$$

(42) $$R=\left(\begin{array}{ccccccccccccccccccccccc} 1 & 1 & 1 & 0 & 0 & 0 & 0 & 0 & 0 & 0 & 0 & 0 & 0 & 0 & 0 & 0 & 0 & 0 & 0 & 0 & 0 & 0 & 0\\ 0 & 0 & 0 & 1 & 1 & 1 & 1 & 1 & 0 & 0 & 0 & 0 & 0 & 0 & 0 & 0 & 0 & 0 & 0 & 0 & 0 & 0 & 0\\ 0 & 0 & 0 & 0 & 0 & 0 & 0 & 0 & 1 & 1 & 1 & 1 & 1 & 0 & 0 & 0 & 0 & 0 & 0 & 0 & 0 & 0 & 0\\ 0 & 0 & 0 & 0 & 0 & 0 & 0 & 0 & 0 & 0 & 0 & 0 & 0 & 1 & 1 & 1 & 1 & 1 & 0 & 0 & 0 & 0 & 0\\ 0 & 0 & 0 & 0 & 0 & 0 & 0 & 0 & 0 & 0 & 0 & 0 & 0 & 0 & 0 & 0 & 0 & 0 & 1 & 1 & 1 & 1 & 1\\ 0 & 0 & 0 & 1 & 0 & 0 & 0 & 0 & 1 & 0 & 0 & 0 & 0 & 1 & 0 & 0 & 0 & 0 & 1 & 0 & 0 & 0 & 0\\ 0 & 0 & 0 & 0 & 1 & 0 & 0 & 0 & 0 & 1 & 0 & 0 & 0 & 0 & 1 & 0 & 0 & 0 & 0 & 1 & 0 & 0 & 0\\ 1 & 0 & 0 & 0 & 0 & 1 & 0 & 0 & 0 & 0 & 1 & 0 & 0 & 0 & 0 & 1 & 0 & 0 & 0 & 0 & 1 & 0 & 0\\ 0 & 1 & 0 & 0 & 0 & 0 & 1 & 0 & 0 & 0 & 0 & 1 & 0 & 0 & 0 & 0 & 1 & 0 & 0 & 0 & 0 & 1 & 0\\ 0 & 0 & 1 & 0 & 0 & 0 & 0 & 1 & 0 & 0 & 0 & 0 & 1 & 0 & 0 & 0 & 0 & 1 & 0 & 0 & 0 & 0 & 1 \end{array}\right).$$

*By removing the first two points from the RBIBD, we can obtain a matrix R. R serves as the parity-check matrix of a (23,14,4,2) AS-LRC. Its minimum distance is *4*, and its code rate is $\frac{14}{23}$. Using the construction in Theorem 5, we can obtain a 10×33 parity-check matrix $H=\left(\begin{array}{cc} R & |I_{10} \end{array}\right)$ and a 23×33 generator matrix $G=\left(\begin{array}{cc} I_{23} & |R^{T} \end{array}\right)$.*

(43) $$G^{T}=\left(\begin{array}{ccccccccccccccccccccccc} 1 & 0 & 0 & 0 & 0 & 0 & 0 & 0 & 0 & 0 & 0 & 0 & 0 & 0 & 0 & 0 & 0 & 0 & 0 & 0 & 0 & 0 & 0\\ 0 & 1 & 0 & 0 & 0 & 0 & 0 & 0 & 0 & 0 & 0 & 0 & 0 & 0 & 0 & 0 & 0 & 0 & 0 & 0 & 0 & 0 & 0\\ 0 & 0 & 1 & 0 & 0 & 0 & 0 & 0 & 0 & 0 & 0 & 0 & 0 & 0 & 0 & 0 & 0 & 0 & 0 & 0 & 0 & 0 & 0\\ 0 & 0 & 0 & 1 & 0 & 0 & 0 & 0 & 0 & 0 & 0 & 0 & 0 & 0 & 0 & 0 & 0 & 0 & 0 & 0 & 0 & 0 & 0\\ 0 & 0 & 0 & 0 & 1 & 0 & 0 & 0 & 0 & 0 & 0 & 0 & 0 & 0 & 0 & 0 & 0 & 0 & 0 & 0 & 0 & 0 & 0\\ 0 & 0 & 0 & 0 & 0 & 1 & 0 & 0 & 0 & 0 & 0 & 0 & 0 & 0 & 0 & 0 & 0 & 0 & 0 & 0 & 0 & 0 & 0\\ 0 & 0 & 0 & 0 & 0 & 0 & 1 & 0 & 0 & 0 & 0 & 0 & 0 & 0 & 0 & 0 & 0 & 0 & 0 & 0 & 0 & 0 & 0\\ 0 & 0 & 0 & 0 & 0 & 0 & 0 & 1 & 0 & 0 & 0 & 0 & 0 & 0 & 0 & 0 & 0 & 0 & 0 & 0 & 0 & 0 & 0\\ 0 & 0 & 0 & 0 & 0 & 0 & 0 & 0 & 1 & 0 & 0 & 0 & 0 & 0 & 0 & 0 & 0 & 0 & 0 & 0 & 0 & 0 & 0\\ 0 & 0 & 0 & 0 & 0 & 0 & 0 & 0 & 0 & 1 & 0 & 0 & 0 & 0 & 0 & 0 & 0 & 0 & 0 & 0 & 0 & 0 & 0\\ 0 & 0 & 0 & 0 & 0 & 0 & 0 & 0 & 0 & 0 & 1 & 0 & 0 & 0 & 0 & 0 & 0 & 0 & 0 & 0 & 0 & 0 & 0\\ 0 & 0 & 0 & 0 & 0 & 0 & 0 & 0 & 0 & 0 & 0 & 1 & 0 & 0 & 0 & 0 & 0 & 0 & 0 & 0 & 0 & 0 & 0\\ 0 & 0 & 0 & 0 & 0 & 0 & 0 & 0 & 0 & 0 & 0 & 0 & 1 & 0 & 0 & 0 & 0 & 0 & 0 & 0 & 0 & 0 & 0\\ 0 & 0 & 0 & 0 & 0 & 0 & 0 & 0 & 0 & 0 & 0 & 0 & 0 & 1 & 0 & 0 & 0 & 0 & 0 & 0 & 0 & 0 & 0\\ 0 & 0 & 0 & 0 & 0 & 0 & 0 & 0 & 0 & 0 & 0 & 0 & 0 & 0 & 1 & 0 & 0 & 0 & 0 & 0 & 0 & 0 & 0\\ 0 & 0 & 0 & 0 & 0 & 0 & 0 & 0 & 0 & 0 & 0 & 0 & 0 & 0 & 0 & 1 & 0 & 0 & 0 & 0 & 0 & 0 & 0\\ 0 & 0 & 0 & 0 & 0 & 0 & 0 & 0 & 0 & 0 & 0 & 0 & 0 & 0 & 0 & 0 & 1 & 0 & 0 & 0 & 0 & 0 & 0\\ 0 & 0 & 0 & 0 & 0 & 0 & 0 & 0 & 0 & 0 & 0 & 0 & 0 & 0 & 0 & 0 & 0 & 1 & 0 & 0 & 0 & 0 & 0\\ 0 & 0 & 0 & 0 & 0 & 0 & 0 & 0 & 0 & 0 & 0 & 0 & 0 & 0 & 0 & 0 & 0 & 0 & 1 & 0 & 0 & 0 & 0\\ 0 & 0 & 0 & 0 & 0 & 0 & 0 & 0 & 0 & 0 & 0 & 0 & 0 & 0 & 0 & 0 & 0 & 0 & 0 & 1 & 0 & 0 & 0\\ 0 & 0 & 0 & 0 & 0 & 0 & 0 & 0 & 0 & 0 & 0 & 0 & 0 & 0 & 0 & 0 & 0 & 0 & 0 & 0 & 1 & 0 & 0\\ 0 & 0 & 0 & 0 & 0 & 0 & 0 & 0 & 0 & 0 & 0 & 0 & 0 & 0 & 0 & 0 & 0 & 0 & 0 & 0 & 0 & 1 & 0\\ 0 & 0 & 0 & 0 & 0 & 0 & 0 & 0 & 0 & 0 & 0 & 0 & 0 & 0 & 0 & 0 & 0 & 0 & 0 & 0 & 0 & 0 & 1\\ 1 & 1 & 1 & 0 & 0 & 0 & 0 & 0 & 0 & 0 & 0 & 0 & 0 & 0 & 0 & 0 & 0 & 0 & 0 & 0 & 0 & 0 & 0\\ 0 & 0 & 0 & 1 & 1 & 1 & 1 & 1 & 0 & 0 & 0 & 0 & 0 & 0 & 0 & 0 & 0 & 0 & 0 & 0 & 0 & 0 & 0\\ 0 & 0 & 0 & 0 & 0 & 0 & 0 & 0 & 1 & 1 & 1 & 1 & 1 & 0 & 0 & 0 & 0 & 0 & 0 & 0 & 0 & 0 & 0\\ 0 & 0 & 0 & 0 & 0 & 0 & 0 & 0 & 0 & 0 & 0 & 0 & 0 & 1 & 1 & 1 & 1 & 1 & 0 & 0 & 0 & 0 & 0\\ 0 & 0 & 0 & 0 & 0 & 0 & 0 & 0 & 0 & 0 & 0 & 0 & 0 & 0 & 0 & 0 & 0 & 0 & 1 & 1 & 1 & 1 & 1\\ 0 & 0 & 0 & 1 & 0 & 0 & 0 & 0 & 1 & 0 & 0 & 0 & 0 & 1 & 0 & 0 & 0 & 0 & 1 & 0 & 0 & 0 & 0\\ 0 & 0 & 0 & 0 & 1 & 0 & 0 & 0 & 0 & 1 & 0 & 0 & 0 & 0 & 1 & 0 & 0 & 0 & 0 & 1 & 0 & 0 & 0\\ 1 & 0 & 0 & 0 & 0 & 1 & 0 & 0 & 0 & 0 & 1 & 0 & 0 & 0 & 0 & 1 & 0 & 0 & 0 & 0 & 1 & 0 & 0\\ 0 & 1 & 0 & 0 & 0 & 0 & 1 & 0 & 0 & 0 & 0 & 1 & 0 & 0 & 0 & 0 & 1 & 0 & 0 & 0 & 0 & 1 & 0\\ 0 & 0 & 1 & 0 & 0 & 0 & 0 & 1 & 0 & 0 & 0 & 0 & 1 & 0 & 0 & 0 & 0 & 1 & 0 & 0 & 0 & 0 & 1 \end{array}\right)$$

(44) $$H^{T}=\left(\begin{array}{cccccccccc} 1 & 0 & 0 & 0 & 0 & 0 & 0 & 1 & 0 & 0\\ 1 & 0 & 0 & 0 & 0 & 0 & 0 & 0 & 1 & 0\\ 1 & 0 & 0 & 0 & 0 & 0 & 0 & 0 & 0 & 1\\ 0 & 1 & 0 & 0 & 0 & 1 & 0 & 0 & 0 & 0\\ 0 & 1 & 0 & 0 & 0 & 0 & 1 & 0 & 0 & 0\\ 0 & 1 & 0 & 0 & 0 & 0 & 0 & 1 & 0 & 0\\ 0 & 1 & 0 & 0 & 0 & 0 & 0 & 0 & 1 & 0\\ 0 & 1 & 0 & 0 & 0 & 0 & 0 & 0 & 0 & 1\\ 0 & 0 & 1 & 0 & 0 & 1 & 0 & 0 & 0 & 0\\ 0 & 0 & 1 & 0 & 0 & 0 & 1 & 0 & 0 & 0\\ 0 & 0 & 1 & 0 & 0 & 0 & 0 & 1 & 0 & 0\\ 0 & 0 & 1 & 0 & 0 & 0 & 0 & 0 & 1 & 0\\ 0 & 0 & 1 & 0 & 0 & 0 & 0 & 0 & 0 & 1\\ 0 & 0 & 0 & 1 & 0 & 1 & 0 & 0 & 0 & 0\\ 0 & 0 & 0 & 1 & 0 & 0 & 1 & 0 & 0 & 0\\ 0 & 0 & 0 & 1 & 0 & 0 & 0 & 1 & 0 & 0\\ 0 & 0 & 0 & 1 & 0 & 0 & 0 & 0 & 1 & 0\\ 0 & 0 & 0 & 1 & 0 & 0 & 0 & 0 & 0 & 1\\ 0 & 0 & 0 & 0 & 1 & 1 & 0 & 0 & 0 & 0\\ 0 & 0 & 0 & 0 & 1 & 0 & 1 & 0 & 0 & 0\\ 0 & 0 & 0 & 0 & 1 & 0 & 0 & 1 & 0 & 0\\ 0 & 0 & 0 & 0 & 1 & 0 & 0 & 0 & 1 & 0\\ 0 & 0 & 0 & 0 & 1 & 0 & 0 & 0 & 0 & 1\\ 1 & 0 & 0 & 0 & 0 & 0 & 0 & 0 & 0 & 0\\ 0 & 1 & 0 & 0 & 0 & 0 & 0 & 0 & 0 & 0\\ 0 & 0 & 1 & 0 & 0 & 0 & 0 & 0 & 0 & 0\\ 0 & 0 & 0 & 1 & 0 & 0 & 0 & 0 & 0 & 0\\ 0 & 0 & 0 & 0 & 1 & 0 & 0 & 0 & 0 & 0\\ 0 & 0 & 0 & 0 & 0 & 1 & 0 & 0 & 0 & 0\\ 0 & 0 & 0 & 0 & 0 & 0 & 1 & 0 & 0 & 0\\ 0 & 0 & 0 & 0 & 0 & 0 & 0 & 1 & 0 & 0\\ 0 & 0 & 0 & 0 & 0 & 0 & 0 & 0 & 1 & 0\\ 0 & 0 & 0 & 0 & 0 & 0 & 0 & 0 & 0 & 1 \end{array}\right)$$

*Due to space limitations, we give the transposed form of G and H. We obtain a (33,23,5,2) IS-LRC C with nonuniform localities. The minimum distance of this code is d(C)=3, which attains the bound in (3). $R\left(C\right)=\frac{23}{33}$. The first message symbol in the code has 2 recovery sets: $R_{1}\left(1\right)=\left\{2,3,24\right\}$ and $R_{2}\left(1\right)=\left\{6,11,16,21,31\right\}$, in which each repair set contains one parity symbol. When this information symbol is lost, we can recover it by XORing all symbols indexed by $R_{1}\left(1\right)$ or $R_{2}\left(1\right)$.*

## 4. Discussion

In this section, we compare the parameters of our constructions with those of some related works. Table 1 gives the parameters of all constructions proposed in this paper. In Table 2 and Table 3, we give the parameters of some locally repairable codes with information-symbol (r,t)-availability from this paper and related constructions.

First, we compare our constructions with the codes constructed in [15] with all-symbol (r,t)-availability. Since a $\left(\hat{v},\hat{k},1\right)$-BIBD is also a $\left(\hat{v},\hat{k},1\right)$-PBD, we can use our constructions in Section 3.1 to obtain an $\left(n=\hat{b},k,r=\hat{r}-1,t=\hat{k}\right)$ LRC with all-symbol availability, where d(C)≥t+1 and $R\left(C\right)\geq 1-\frac{t}{r+1}$. This parameter set includes the first code constructed in [15]. The construction of an $\left(n=\left(r+1\right)\frac{\hat{v}}{\hat{k}},k,r\in \left[t,\hat{r}-1\right],t=\hat{k}-1\right)$ LRC in Theorem 2 from a $\left(\hat{v}-1,\hat{r},\left\{\hat{k},\hat{k}-1\right\},1\right)$-PBD leads to multiple choices of *r* for fixed *t*, and the parameters of this construction cannot be derived from [15]. Moreover, the codes from Section 3.1 and Section 3.2 give nonuniform locality or nonuniform availability LRCs, and we can obtain a distance-optimal (7,3,2,2) LRC with availabilities of 2 and 3 for different coordinates from Theorem 1.

Then, we consider locally repairable codes with information (r,t)-availability. Since a $\left(\hat{v},\hat{k},1\right)$-BIBD is also a $\left(\hat{v},\hat{k},1\right)$-PBD, we can use the codes in Section 3.3 and Section 3.4 to obtain three classes of distance-optimal constructions from BIBDs. These constructions contain the codes from [15]. Moreover, our codes in Theorem 4 give locally repairable codes with nonuniform availability, and our construction in Theorem 5 proposes distance-optimal IS-LRCs with nonuniform locality. The locally repairable codes in this paper contain almost all the codes from [15], except for those derived from Latin squares. Table 2 contains the corresponding properties of these constructions.

Then, we compare our construction with some known distance-optimal locally repairable codes from [6,11,18,24,25]. Locally repairable codes with (r,δ)-locality [6] focus on enhancing the fault tolerance ability of each repair group. In this kind of code, each code symbol has multiple choices of repair groups. However, there is no guarantee that these repair sets are disjoint. On the other hand, locally repairable codes with multiple disjoint recovery sets [11,18,24,25] support parallel reading of hot data.

Su [11] proposed a series of distance-optimal locally repairable codes with information symbol (r,t)-availability from (k,b,c,r) resolvable configurations and an (N+t,k) MDS code or an (N+t−1,k) Gabidulin code. The first kind of construction in [11] gave ${\left(N+\frac{kt}{r},k,r,t\right)}_{q}$ IS-LRCs without all-symbol locality and a minimum distance of N−k+t+1, where q≥N+t,r|k. The second kind of construction in [11] gave distance-optimal ${\left(N+\frac{N}{r}+\left(t-1\right)\frac{kt}{r},k,r,t\right)}_{q^{M}}$ IS-LRCs with all-symbol locality, where M≥N+t−1. Compared to the codes in [11], our distance-optimal constructions in Section 3.3 and Section 3.4 achieve higher code rates and require much smaller field sizes, leading to lower computational and storage overhead. Moreover, the locality of our codes in Section 3.3 and Section 3.4 is *r* for all code symbols, while the code in [11] with all-symbol locality has a field size that is exponential in the code length *n*. Compared to the codes in [11], our distance-optimal codes achieve the smallest finite field size, a higher code rate, and all-symbol locality at the expense of a lower minimum distance (minimum distance of our codes still attains the bound in (3)).

Rawat et al. [8] raised an open question: whether r|k is a necessary condition for (n,k,r,t) locally repairable codes to attain the bound in (3). Zhang et al. [15] and Cai et al. [24] addressed this open question by proposing distance-optimal locally repairable codes, where r∤k. Cai et al. presented a framework for constructing locally repairable codes with optimal distance using (k,R,1)-packings. A (k,R,1)-packing can be viewed as a generalization of a PBD that requires each pair of points can appear at most once in all blocks. Cai et al. gave a series of distance-optimal locally repairable codes from packings when r|kt. The parameters of these constructions are $\left(n=k+\frac{kt}{r},k,r,t\right)$, d=t+1, and $R=\frac{r}{r+t}$. Our constructions yield similar parameters in this case. The authors also gave a series of constructions when r∤kt. The parameters of these constructions are listed in Table 3. Our construction in Theorem 5 yields similar parameters as these constructions when r=3 or r=4, as well as in other cases. For example, in our constructions from Theorem 5, given a $\left(\hat{v},\hat{k},1\right)$-RBIBD, we can obtain an LRC with parameters $(n=\hat{v}-1+t\frac{\hat{v}}{\hat{k}}$, $k=\hat{v}-1,r=\hat{k},t<r)$. We can obtain a construction with the same *r* and *t* as the codes in rows 3 and 4 of Table 3 [24]. When we choose s=v in the construction from Theorem 5, we obtain a construction with the same *r* and *t* as the codes in [24] in the second row of Table 3. Moreover, our distance-optimal constructions in Section 3.4 have higher code rates and smaller field sizes, and all coordinates in our constructions in Section 3.4 have locality *r*. We also addressed the open problem proposed in [8]. Therefore, compared to the construction in [24], the codes in this paper have certain advantages.

Cai et al. [25] also proposed a construction of distance-optimal locally repairable codes with (r,t)-availability from linearized Reed–Solomon codes and Gabidulin codes. Their constructions can attain the bound in (2). The parameters of these constructions are listed in Table 3. The constructions in [25] give ${\left(n=k\left(1+rt\right),k,r,t\right)}_{q}$ LRCs with information (r,t)-availability for arbitrary *r* and *t*. They give the first explicit construction of LRCs with minimum distance to attain the bound in (2). Compared to the codes in [25], our constructions in Section 3.4 have a smaller minimum distance. However, the code rate of the constructions in [25] is $\frac{1}{rt+1}$, and the field size is much larger than 2. The code rate of our constructions is higher than that of the constructions in [25]. In addition, our constructions are binary and distance-optimal, so they are more suitable for deployment in actual distributed storage systems.

In [18], Tan et al. proposed two classes of optimal LRCs with message-symbol (r,t)-availability from linear algebra and partial geometries. Note that some partial geometries are BIBDs, so our constructions in Section 3.3 and Section 3.4 include the constructions from [18] when the chosen partial geometry is a BIBD. Their codes from linear algebra with $\left(n=r^{2}+tr,k=r^{2},r,t\right)$, where t≤r and *r* is a prime, are included in our constructions from an affine plane of order *r*. Their constructions with parameters $\left(n=r^{t}+tr^{t}-1,k=r^{t},r,t\right)$ are covered in our codes when an $\left(r^{t},r,1\right)$-BIBD exists. The codes in [15] can also yield the parameters mentioned above. Therefore, our codes include part of the constructions from [18]. Moreover, our constructions can yield binary distance-optimal codes when r∤k, while the constructions in [11,18] cannot.

## 5. Conclusions

This paper presents a comprehensive framework for constructing locally repairable codes (LRCs) using pairwise balanced designs (PBDs). In this paper, we propose LRCs with all-symbol locality and nonuniform availability using PBDs. These constructions include previously developed codes from [15]. In some cases, this construction can achieve distance optimality under the bounds in (2) and (3). We also introduce a method for constructing LRCs with nonuniform localities and all-symbol availability.

We construct locally repairable codes with uniform locality and nonuniform availability from PBDs. When the chosen PBD is a BIBD, our construction is distance-optimal and rate-optimal for t=2. This construction contains the codes from [15].

Moreover, we develop distance-optimal LRCs with nonuniform localities and message (r,t)-availability, addressing the open question proposed in [8]. Our construction contains some codes proposed in [15,18,24], and its code rate outperforms that of the codes in [11,24,25] with a much smaller field size, demonstrating superiority over state-of-the-art constructions in terms of code rate, finite field size, and repair efficiency. Our construction can also produce codes with parameters not contained in [11,15,18,24,25].

Through detailed comparisons with existing works, including those by Su et al. [11], Cai et al. [24,25], Zhang et al. [15], and Tan et al. [18], we have shown that our constructions either subsume or outperform prior codes in terms of code rate, minimum distance, and computational complexity. Moreover, our binary implementations make these constructions highly suitable for practical deployment in distributed storage systems. Overall, the results in this paper provide a unified and generalized approach for constructing high-performance locally repairable codes, with significant theoretical and practical implications.

## Figures and Tables

**Table 1:** Parameters of all constructions proposed in this paper.

Ref.	Field Size	Underlying Structures	Parameters	Minimum Distance d(C)	Rate R(C)	Special Properties
Theorem 1	2	$\left(\hat{v},\hat{K},1\right)$-PBD	$n=\hat{b},k$, $r=\hat{r}-1,t={\hat{k}}_{\min}$	d(C)≥t+1	$R\left(C\right)\geq 1-\frac{{\hat{k}}_{\mathrm{avg}}}{r}$	Nonuniform availability
Theorem 1, special case	2	(6,{2,3},1)-PBD	7,3,2,2	4, distance-optimal	$\frac{3}{7}$	Nonuniform availability
Remark 1	2	$\left(\hat{v},\hat{k},1\right)$-BIBD	$n=\hat{b},k$, $r=\hat{r}-1,t=\hat{k}$	d(C)≥t+1	$R\left(C\right)\geq 1-\frac{t}{r+1}$	None
Theorem 2	2	$\left(\hat{v}-1,\hat{r},\left\{\hat{k},\hat{k}-1\right\},1\right)$-PBD from RBIBD	$n=\left(r+1\right)\frac{\hat{v}}{\hat{k}},k$, $r\in \left[t,\hat{r}-1\right],t=\hat{k}-1$	d(C)≥t+1	$R\left(C\right)\geq 1-\frac{\left(r+1\right)}{\left(t+1\right)}\frac{\left(\hat{v}-1\right)}{\hat{v}}$	Nonuniform availability
Theorem 3	2	PBD from $\left(\hat{v},\hat{b},\hat{r},\hat{k},1\right)$-RBIBD	$n=\hat{v}-1,k$, $r=\hat{k}-1,t\leq r$	d(C)≥t+1	$R\left(C\right)\geq 1-\frac{t(n+1)}{(r+1)n}-\frac{t-1}{n}$	Nonuniform locality
Remark 2	2	$\left(\hat{v},\hat{b},\hat{r},\hat{k},1\right)$-RBIBD	$n=\hat{v},k$, $r=\hat{k}-1,t\leq \hat{k}$	d(C)≥t+1	$R\left(C\right)\geq 1-\frac{t}{\left(r+1\right)}-\frac{t-1}{n}$	None
Theorem 4	2	$\left(\hat{v},\hat{K},1\right)$-PBD	$n=\hat{b}+\hat{v},k=\hat{b}$, $r=\hat{r},t={\hat{k}}_{\min}$	d(C)≥t+1	$R\left(C\right)=\frac{\hat{b}}{\hat{b}+\hat{v}}$	Nonuniform availability
Remark 3	2	$\left(\hat{v},\hat{b},\hat{r},\hat{k},1\right)$-BIBD	$n=\hat{v}+\hat{b},k=\hat{b}$, $r=\hat{r},t=\hat{k}$	d(C)=t+1, distance-optimal	$R\left(C\right)=\frac{r}{r+t}$, rate-optimal when t=2	None
Remark 3	2	$\left(\hat{v},\hat{b},\hat{r},\hat{k},1\right)$-BIBD	$n=\hat{v}+\hat{b},k=\hat{v}$, $r=\hat{k},t=\hat{r}$	d(C)=t+1, distance-optimal	$R\left(C\right)=\frac{r}{r+t}$, rate-optimal when t=2	None
Remark 4	2	$\left(\hat{v},\hat{b},\hat{r},\hat{k},1\right)$-RBIBD	$n=\hat{v}+t\frac{\hat{v}}{k}$, $k=\hat{v},r=\hat{k},t\leq \hat{r}$	d(C)=t+1, distance-optimal	$R\left(C\right)=\frac{r}{r+t}$, rate-optimal when t=2	None
Theorem 5	2	$\left(\hat{v},\hat{b},\hat{r},\hat{k},1\right)$-RBIBD	$n=\hat{v}-s+t\frac{\hat{v}}{\hat{k}}$, $k=\hat{v}-s,s+1\leq r=\hat{k},t<\frac{r}{s}$	d(C)=t+1, distance-optimal	$R\left(C\right)=\frac{\hat{v}-s}{\hat{v}-s+t\frac{\hat{v}}{\hat{k}}}$	Nonuniform locality

**Table 2:** Some known locally repairable codes with information-symbol (r,t)-availability, part 1.

Ref.	Field Size	Underlying Structures	Parameters	Minimum Distance d(C)	Rate R(C)	Special Properties
Theorem 4	2	$\left(\hat{v},\hat{K},1\right)$-PBD	$n=\hat{b}+\hat{v},k=\hat{b}$, $r=\hat{r},t={\hat{k}}_{\min}$	d(C)≥t+1	$R\left(C\right)=\frac{\hat{b}}{\hat{b}+\hat{v}}$	Nonuniform availability, all-symbol locality, r∤kt
Remark 3	2	$\left(\hat{v},\hat{b},\hat{r},\hat{k},1\right)$-BIBD	$n=\hat{v}+\hat{b},k=\hat{b}$, $r=\hat{r},t=\hat{k}$	d(C)=t+1, distance-optimal	$R\left(C\right)=\frac{r}{r+t}$, rate-optimal when t=2	All-symbol locality, r|kt
Remark 3	2	$\left(\hat{v},\hat{b},\hat{r},\hat{k},1\right)$-BIBD	$n=\hat{v}+\hat{b},k=\hat{v}$, $r=\hat{k},t=\hat{r}$	d(C)=t+1, distance-optimal	$R\left(C\right)=\frac{r}{r+t}$, rate-optimal when t=2	All-symbol locality, r|kt
[15]	2	$\left(\hat{v},\hat{b},\hat{r},\hat{k},1\right)$-BIBD	$n=\hat{v}+\hat{b},k=\hat{b}$, $r=\hat{r},t=\hat{k}$	d(C)=t+1, distance-optimal	$R\left(C\right)=\frac{r}{r+t}$, rate-optimal when t=2	All-symbol locality, r|kt
[15]	2	$\left(\hat{v},\hat{b},\hat{r},\hat{k},1\right)$-BIBD	$n=\hat{v}+\hat{b},k=\hat{v}$, $r=\hat{k},t=\hat{r}$	d(C)=t+1, distance-optimal	$R\left(C\right)=\frac{r}{r+t}$, rate-optimal when t=2	All-symbol locality, r|kt
Remark 4	2	$\left(\hat{v},\hat{b},\hat{r},\hat{k},1\right)$-RBIBD	$n=\hat{v}+t\frac{\hat{v}}{k}$, $k=\hat{v},r=\hat{k},t\leq \hat{r}$	d(C)=t+1, distance-optimal	$R\left(C\right)=\frac{r}{r+t}$, rate-optimal when t=2	All-symbol locality, r|kt
[15]	2	$\left(\hat{v},\hat{b},\hat{r},\hat{k},1\right)$-RBIBD	$n=\hat{v}+t\frac{\hat{v}}{k}$, $k=\hat{v},r=\hat{k},t\leq \hat{r}$	d(C)=t+1, distance-optimal	$R\left(C\right)=\frac{r}{r+t}$, rate-optimal when t=2	All-symbol locality, r|kt
Theorem 5	2	$\left(\hat{v},\hat{b},\hat{r},\hat{k},1\right)$-RBIBD	$n=\hat{v}-s+t\frac{\hat{v}}{\hat{k}}$, $k=\hat{v}-s,s+1\leq r=\hat{k},t<\frac{r}{s}$	d(C)=t+1, distance-optimal	$R\left(C\right)=\frac{\hat{v}-s}{\hat{v}-s+t\frac{\hat{v}}{\hat{k}}}$	Nonuniform locality, all-symbol locality,r∤kt
[11]	≥N+t	(N+t,k) MDS code, (k,b,c,r) resolvable configuration	$\left(N+\frac{kt}{r},k,r,t\right)$	d(C)=N−k+t+1, distance-optimal	$R\left(C\right)=\frac{k}{N+\frac{kt}{r}}$	Without all-symbol locality, r|kt
[11]	≥$q^{N+t-1}$	(N+t−1,k) Gabidulin code, (k,b,c,r) resolvable configuration	$\left(N+\frac{N}{r}+\left(t-1\right)\frac{kt}{r},k,r,t\right)$	$d\left(C\right)=N+\frac{N}{r}-k-\frac{k}{r}+t+1$, distance-optimal	$R\left(C\right)=\frac{k}{N+\frac{N}{r}+\left(t-1\right)\frac{kt}{r}}$	All-symbol locality, r|kt

**Table 3:** Some known locally repairable codes with information-symbol (r,t)-availability, part 2.

Ref.	Field Size	Underlying Structures	Parameters	Minimum Distance d(C)	Rate R(C)	Special Properties
[24]	2	(k,r,1)-packing	$n=k+\frac{kt}{r},k,r,t$	d(C)=t+1, distance-optimal	$R\left(C\right)=\frac{r}{r+t}$, rate-optimal when t=2	All-symbol locality, r|kt
[24]	≥k+d−1	(rm,r,1)-packing,(k+d−1,k,d)-MDS code	n=k+tm−t+d−1,k=rm−v,r,t	d(C)=d, distance-optimal	$R\left(C\right)=\frac{k}{k+tm-t+d-1}$	Without all-symbol locality, v∈[m−1],r,t∈[m], r∤kt
[24]	≥k+d−1	$\left(k,\left\{p^{a},p^{a}-1\right\},1\right)$-packing,(k+d−1,k,d)-MDS code	$n=k+\left(\left\lceil \frac{k}{r}\right\rceil -1\right)t+d-1,k,r=p^{a},t$	d(C)=d, distance-optimal	$R\left(C\right)=\frac{k}{k+(\left\lceil \frac{k}{r}\right\rceil -1)t+d-1}$	Without all-symbol locality, *p* is a prime, r∤kt
[24]	≥k+d−1	(k,{r,r−1},1)-packing,(k+d−1,k,d)-MDS code	$n=k+(\left\lceil \frac{k}{r}\right\rceil -1)t+d-1,k,r=t$	d(C)=d, distance-optimal	$R\left(C\right)=\frac{k}{k+(\left\lceil \frac{k}{r}\right\rceil -1)t+d-1}$	Without all-symbol locality, r=3,4, r∤kt
[25]	≥$q_{1}^{k(1+(r-1)t)}$	Gabidulin code	n=k(1+rt),k,r,t	$d\left(C\right)=n-k-\left\lceil \frac{t(k-1)+1}{t(r-1)+1}\right\rceil +2$	$R\left(C\right)=\frac{1}{1+rt}$	$q_{1}\geq r+1$, all-symbol locality
[25]	≥$q_{1}^{1+(r-1)t}$	Linearized Reed–Solomon code	n=k(1+rt),k,r,t	$d\left(C\right)=n-k-\left\lceil \frac{t(k-1)+1}{t(r-1)+1}\right\rceil +2$	$R\left(C\right)=\frac{1}{1+rt}$	$q_{1}\geq k+1$, all-symbol locality
[18]	2	Linear algebra	$n=r^{2}+tr,k=r^{2},r,t$	d(C)=t+1, distance-optimal	$R\left(C\right)=\frac{r}{r+t}$, rate-optimal when t=2	*r* is a prime, t≤r, r|k
[18]	2	Linear algebra	$n=r^{t}+tr^{t-1},k=r^{t},r,t$	d(C)=t+1, distance-optimal	$R\left(C\right)=\frac{r}{r+t}$, rate-optimal when t=2	r|k
[18]	2	$\hat{b}\times \hat{v}$ incidence matrix of a partial geometry PG(s+1,u+1)	$n=\hat{v}+\hat{b},k=\hat{b},r=u+1,t=s+1$	d(C)=t+1, distance-optimal	$R\left(C\right)=\frac{r}{r+t}$, rate-optimal when t=2	r|kt
[18]	2	Dual code of codes from PG(s+1,u+1)	$n=\hat{v}+\hat{b},k=\hat{v},r=s+1,t=u+1$	d(C)=t+1, distance-optimal	$R\left(C\right)=\frac{r}{r+t}$, rate-optimal when t=2	r|kt

## Data Availability

Data are contained within the article.
